# Exploring the use of routinely-available, retrospective data to study the association between malaria control scale-up and micro-economic outcomes in Zambia

**DOI:** 10.1186/s12936-016-1665-z

**Published:** 2017-01-04

**Authors:** Alison Comfort, Anthony Leegwater, Sharon Nakhimovsky, Henry Kansembe, Busiku Hamainza, Benson Bwalya, Martin Alilio, Ben Johns, Lauren Olsho

**Affiliations:** 1 Health Finance and Governance Project, Abt Associates Inc., Bethesda, USA; 2 National Malaria Control Centre, Zambia Ministry of Health, Lusaka, Zambia; 3 President’s Malaria Initiative, Washington, D.C., USA; 4 International Health Division, Abt Associates Inc., Lusaka, Zambia; 5 U.S. Health Division, Abt Associates Inc., Cambridge, USA

**Keywords:** Malaria, Malaria control, Micro-economic outcomes, Routine survey data, Propensity score, Scale-up

## Abstract

**Background:**

Country-level evidence on the impact of malaria control on micro-economic outcomes is vital for mobilizing domestic and donor resources for malaria control. Using routinely available survey data could facilitate this investigation in a cost-efficient way.

**Methods:**

The authors used Malaria Indicator Surveys (MIS) and Living Conditions Monitoring Survey (LCMS) data from 2006 to 2010 for all 72 districts in Zambia to relate malaria control scale-up with household food spending (proxy for household well-being), educational attainment and agricultural production. The authors used two quasi-experimental designs: (1) a generalized propensity score for a continuous treatment variable (defined as coverage from owning insecticide-treated bed nets and/or receipt of indoor residual spraying); and, (2) a district fixed effects model to assess changes in the outcome relative to changes in treatment pre-post scale-up. The unit of analysis was at district level. The authors also conducted simulations post-analysis to assess statistical power.

**Results:**

Micro-economic outcomes increased (33% increase in food spending) concurrently with malaria control coverage (62% increase) from 2006 to 2010. Despite using data from all 72 districts, both analytic methods yielded wide confidence intervals that do not conclusively link outcomes and malaria control coverage increases. The authors cannot rule out positive, null or negative effects. The upper bound estimates of the results show that if malaria control coverage increases from 60 to 70%, food spending could increase up to 14%, maize production could increase up to 57%, and years of schooling could increase up to 0.5 years. Simulations indicated that the generalized propensity score model did not have good statistical power.

**Conclusion:**

While it is technically possible to use routinely available survey data to relate malaria control scale-up and micro-economic outcomes, it is not clear from this analysis that meaningful results can be obtained when survey data are highly aggregated. Researchers in similar settings should assess the feasibility of disaggregating existing survey data. Additionally, large surveys, such as LCMS and MIS, could incorporate data on both malaria coverage and household expenditures, respectively.

**Electronic supplementary material:**

The online version of this article (doi:10.1186/s12936-016-1665-z) contains supplementary material, which is available to authorized users.

## Background

According to the World Health Organization (WHO), investment in malaria strategies needs to increase to meet the new global malaria targets for 2030 [1]. There is a substantial and growing body of evidence demonstrating the effectiveness of malaria control interventions in reducing malaria morbidity and mortality, as well as the benefits of malaria control for health systems [2–10]. However, less is known about the micro-economic impact of such interventions. Effective malaria control can reduce the incidence of malaria, reducing disruption to household economic activities and also reducing household resources allocated to malaria care-seeking [11]. As a result, investment in malaria control could lead to lower rates of work and school absenteeism, improved worker productivity, higher household income, and greater spending on key household commodities. Country-level evidence on the impact of malaria control scale-up strategies on micro-economic outcomes would provide a more holistic picture of the benefits of malaria control and better inform ministries of health and finance, donors, private sector actors, researchers, and other stakeholders to make decisions about allocating resources for malaria.

Studies using experimental and quasi-experimental approaches have explored the impact of malaria control on certain key micro-economic outcomes, especially education. However, many of these studies use a short-term follow-up period and are not necessarily generalizable to national level, meaning there is a lack information on the impact of malaria control over a longer period of time at the country level. Notably, a prospective randomized controlled trial (RCT) conducted in a rural area of Zambia’s Southern Province, found a statistically significant increase in the productivity of farmers (a nearly 15% higher value of agricultural output, given free insecticide-treated nets (ITNs) compared to farmers with access to partially subsidized ITNs through loans [11]). Another RCT in India concluded that offering micro-loans for ITNs led to a reduction of 2 days in lost work or school absenteeism and a decrease of 269 rupees (31%) in medical costs due to malaria over 6 months, relative to the control group [12]. An earlier RCT in Kenya found that ITNs correlated with a reduction of $0.25 in health care expenditure over a 2-week period for children under 5 years old, as well as a statistically insignificant reduction of 0.5 days in household time lost due to caring for sick children [13]. A recent retrospective study in Uganda looked at the long-term, micro-economic benefits of a malaria eradication programme more than a half-century ago in southwestern Uganda. The authors concluded that this programme boosted primary school completion for females, improved educational attainment by half a year for both sexes, and increased the likelihood of male wage labour by nearly 40% [14]. Other studies explore the effects of malaria control interventions on educational outcomes [15–19]. In general, the evidence related to educational outcomes has been mixed.

This study complements existing literature through its national scope, by employing a multi-year study period and by utilizing commonly available malaria and micro-economic data in Zambia to assess the relationship between malaria control scale-up and micro-economic outcomes. The authors considered several potential evaluation methods to answer this research question. A RCT was eschewed for a number of reasons, including costs, political feasibility given the need to vary access to the malaria control intervention by treatment group, and an extended time frame for study. Instead, a quasi-experimental design, drawing on retrospective data, was chosen. The authors purposely chose to use secondary, retrospective data to provide an example of how readily available, high-quality, low-cost data might be used to answer policy-relevant questions using a quasi-experimental design. After considering several quasi-experimental approaches, the authors opted for using both a fixed effects model, based on a difference in differences (DD) approach, and a generalized propensity score (GPS) model because they best suited the data available (one pre- and one post-scale-up time period) and highlighted the effects of scale-up of the intervention (done using measurable criteria such as malaria incidence).

The study data covered the period 2006–2010, when the Government of the Republic of Zambia and cooperating partners substantially scaled up malaria control efforts. The authors tested whether the scale-up of malaria control activities in Zambia is associated with food consumption, total household consumption, schooling attendance, and agricultural production. This study provides an illuminating example of a resource-conscious approach that marries commonly available data with a quasi-experimental design and has potential relevance for countries and researchers interested in assessing the micro-economic effects of malaria control efforts. Ultimately, this study places malaria control strategies within the broader context of poverty alleviation, serving to inform stakeholder decision-making. The authors proceed by discussing the methods employed for this study, the analytical results, and discuss the lessons learned and conclusions from this research endeavour.

## Methods

### Site selection

This study was conducted in Zambia because it has historically high malaria parasite infection prevalence but underwent a rapid scale-up of malaria control interventions starting in 2006. The analysis focuses specifically on the period from 2006 to 2010 because micro-economic data are available for these two time points. During this period, coverage of indoor residual spraying (IRS) gradually increased from eight districts in 2005, to 15 in 2006, 36 in 2007, and by 2010 54 out of a total of 72 districts in Zambia received IRS. Mass distribution of ITNs began in 2005 and peaked in 2007. By 2008, fewer ITNs were distributed because most of the existing need had been met [20].

Despite the scale-up of these malaria control strategies, malaria remains one of the leading causes of morbidity and mortality in Zambia, with 3257 deaths attributed to malaria in 2013 [10]. In 2010, malaria incidence was 330 per 1000 people per year for all ages and 897 for children under the age of five, while fatality rates reached 34 per 1000 hospital admissions per year [21]. Also, while transmission rates in some districts (e.g., those in Lusaka) reached very low levels by 2010, in others they remained low or moderate (e.g., those in Central, Western, and Southern Provinces), or even moderate to high (e.g., those in Eastern, Northern, Muchinga, and Luapula Provinces) [22]. The authors attempted to exploit this variation in malaria incidence and malaria control coverage by district to identify the relationship between malaria control scale-up and micro-economic outcomes.

### Logic model

**Fig. 1:**
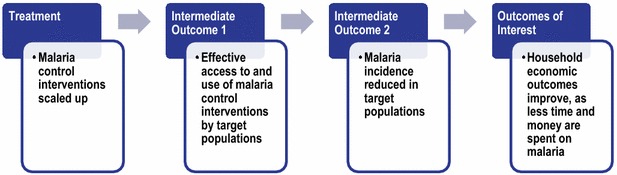
Logical pathway connecting malaria control scale-up with household economic status

This study tests the hypothesis that malaria control scale-up affects micro-economic outcomes at household level through the following intermediate pathway (Fig. 1). The treatment variable represents ownership of ITNs and/or receipt of IRS. However, ownership of ITNs can provide effective protection against malaria only if ITNs are in fact used. For this reason, the first intermediate step in the logical pathway is defined as ‘effective access to and use of malaria control interventions’, which represents use of ITNs and/or receipt of IRS. The expectation is that effective malaria control coverage should reduce the incidence and severity of malaria episodes, the second intermediate step.

In turn, a reduction in the incidence and severity of malaria episodes could improve household economic indicators in various ways. Fewer malaria episodes could reduce the direct costs of malaria by reducing out-of-pocket spending on treatment, freeing up money for other uses such as spending on food and education. Although the Government of Zambia abolished formal user fees at public health facilities in 2006, poorer households may still be burdened by informal costs at point of care, fees for care-seeking at private facilities, and/or transportation costs. Fewer malaria episodes may also reduce the indirect costs associated with illness. These may include time spent seeking treatment or time spent caring for sick household members, allowing the household members to engage in productive agricultural economic activities, such as planting, harvesting, fishing, or attending school for school-age children. Over time, fewer malaria episodes may empower households to make long-term financial decisions around savings, borrowing and investments in productive assets.

### Data sources and measures

Table 1 summarizes the key secondary data sources and variable definitions used in this study. Each measure captures variables associated with a step in the logical pathway (Fig. 1). Variables are aggregated up to the district level and matched by district across the datasets.

**Table 1:** Key secondary data sources and measures

Data	Link to causal pathway (Fig. 1)	Source	Level of data aggregation	Year and frequency of source	Definition of variables in model from data source
Ownership of ITN and/or receipt of IRS	Primary treatment variable	MIS	Household	2006, 2008, and 2010	Per cent of households by district that own at least one ITN/LLIN and/or received IRS in previous 12 months
Use of ITNs/LLINs	Intermediate treatment variable	MIS	Household	2006, 2008, and 2010	Per cent of household members by district who slept under an ITN/LLIN previous night
Use of ITNs/LLINs and/or receipt of IRS	Intermediate treatment variable	MIS	Household	2006, 2008, and 2010	Constructed variable combining ‘use of ITNs/LLINs’ with ‘receipt of IRS’ (since the variable may exceed 100%, the authors capped the variable at 100% in the main analysis but tested other definitions in robustness checks. Note that unlike the Ownership variable, the authors cannot control for overlap between households that use ITNs and are in sprayed dwellings) Per cent of household members by district who slept under an ITN/LLIN previous night and/or received IRS in the previous 12 months
Economic outcomes	Outcome	LCMS	Household	December 2006, February 2010	Household monthly food expenditure Total household monthly expenditure Per cent of school-age children currently in school Years of education attained among school-aged individuals or older Household’s annual production of maize, nuts, potatoes, and cassava
District-level characteristics	Control variables	LCMS	Household	December 2006 February 2010	Average number of children 18 years or younger in the household Average number of children 5 years or younger in the household Average household size Per cent of workers and those looking for work by business type Per cent of household heads who have completed at least primary education Per cent of households with female household head Per cent of households in rural areas Household monthly food expenditure Total household monthly expenditure Per cent of school-age children currently in school Years of education among school-aged individuals or older Household’s annual production of maize, nuts, potatoes, and cassava
		MIS	Household	2006, 2008, and 2010	Prevalence of malaria parasite infection among children 5 years and under
		HMIS	District	Quarterly data for 2005–2008; monthly data 2009–2010 Data come for all hospitals, health centres, and health posts	Total number of outpatient malaria cases (confirmed and clinical) for children and adults by district
		NASA SRTM	GIS 90 m	2008	Elevation by district (mean, median, minimum, maximum, and standard deviation)
		CGIAR-CSI [36]	GIS 0.25°	Annual per agricultural season	Rainfall inches per growing season (October of prior year to September of current year)

*MIS* Malaria Indicator Survey, *NMCC* National Malaria Control Centre, *HMIS* health management information system, *LCMS* Living Conditions Monitoring Survey, *NASA* National Aeronautics and Space Administration, *SRTM* Shuttle Radar Topography Mission, *CGIAR-CSI* Consortium for Spatial Information, *LLIN* long-lasting insecticide-treated net

The primary treatment indicator is defined as the per cent of households who own at least one ITN or long-lasting insecticide-treated net (LLIN) and/or received IRS in the 12 months prior to the survey and come from the Malaria Indicator Survey (MIS). The treatment measure for 2010 can be interpreted as the accumulation of malaria control efforts over the period 2006–2010, since bed nets last more than 1 year and at least some ITNs distributed in earlier years will still provide protection in 2010. The intermediate treatment indicator, which measures effective malaria control coverage, is defined as the per cent of household members who slept under an ITN/LLIN the previous night and/or who received IRS.^1^ The authors chose to use MIS data for the treatment measure rather than National Malaria Control Centre (NMCC) data because the MIS data were deemed more reliable to measure ownership of ITNs and control for overlap in households that own both ITNs and live in sprayed dwellings. Distribution data from the NMCC cannot be readily translated into household level ownership measures.

It is important to highlight that the measures of effective malaria control coverage do not include non-vector control interventions, such as use of artemisinin-based combination therapy and distribution of rapid diagnostic tests to health centres. While these interventions are important for effective diagnosis and treatment of malaria, they are endogenous to malaria prevention efforts (ITN ownership and IRS), meaning that they are part of the intermediate pathway linking ITN ownership/use and IRS with micro-economic outcomes. More specifically, if households adopt effective malaria vector control strategies, the incidence of malaria will decrease as will the need to seek diagnosis and treatment at health facilities. For this reason, the authors do not control for these variables in the model since they are indirectly affected by the treatment variable.

The micro-economic outcomes of interest came from the Living Conditions Monitoring Survey (LCMS) conducted in 2006 and 2010. The primary outcome was monthly household spending on food. As a robustness check, the authors dropped observations for which food spending was in the 99th percentile and made up 60% or more of total monthly spending, since these appeared to be outliers. The authors also investigated other outcomes related to spending, education and agricultural production (Table 1) and conducted additional analyses looking at household borrowing, medical spending, education spending, and wage labour.

A broad set of control variables were included in all analyses. These included all LCMS outcome variables measured at baseline and other key demographic and socio-economic indicators from the LCMS listed in Table 1. In addition, the authors also controlled for elevation, rainfall level during growing season, prevalence of malaria parasite infection among children under 5 years of age, and number of confirmed and unconfirmed outpatient malaria cases.

Data from these sources have several important limitations. First, the treatment variables may be measured with substantial imprecision because the MIS sampling was not intended to produce representative estimates at the district level. The authors dealt with this limitation by using Efron robust standard errors in both the fixed effects and the ordinary least squares model to account for the potential influence of individual district observations on the overall regression plane [23]. The authors did not include this adjustment in the propensity score model because it did not affect the results. Secondly, the MIS data did not include observations for certain districts. To maximize the sample size, the authors imputed missing values for the treatment variable using the average of five datasets imputed by Stata’s multiple imputation command. For the malaria infection prevalence variable, which also comes from the MIS, the authors used a dummy variable adjustment to account for missing observations in baseline values [24].

The LCMS data for the outcome variables also had some limitations. The expenditure aggregates in the 2006 and 2010 LCMS were not adjusted for differences in regional cost of living [25]. The 2006 LCMS questionnaire used by enumerators excluded some own-produced food items that can account for a substantial portion of household consumption in Zambia; this made it difficult to distinguish between zero consumption and missing data. The portion of the 2010 LCMS questionnaire on consumption was revised to address these limitations [25], but the differences in methods between baseline and endline may still introduce bias into the analysis. Finally, the authors had hoped to use both 2006 and 2010 LCMS agricultural production data in the analysis. However, after delays and difficulties in obtaining the 2006 agricultural production data, the summary statistics were substantially different from the 2006 LCMS report. The authors decided not to include the 2006 data obtained and, therefore, the 2006 agricultural production data were excluded from the fixed effects analysis.

The data from the health management information system (HMIS) on outpatient malaria cases also has important limitations. As Ashraf et al. [26] document, the degree of missing data and general data entry error changed over the studied time period due to the transition from a paper-based to an electronic system in 2009. This introduced variability which could not be controlled for in the model. The reliability of the HMIS outpatient malaria data was also affected by concurrent changes in the health system: for example, the scale-up of rapid diagnostic tests used at primary health centres and developments in clinical and reporting practices. These changes may have caused the number of reported malaria cases to fall, making it difficult to isolate real impact on the health status of the population.

### Analytical approach

#### Models

The authors first ran ordinary least squares (OLS) analyses to better understand the correlation between the scale-up over time of malaria control efforts and the micro-economic outcomes. These results were intended to highlight any targeting of interventions and potential selection effects in terms of which areas were targeted earlier.

For the main analysis, the authors considered several quasi-experimental approaches, including regression discontinuity, DD, propensity score matching (PSM), and instrumental variable approaches. Two quasi-experimental designs were chosen. The first approach used the GPS, developed as an extension to PSM to be applied for continuous treatment variables [27]. The second approach used a fixed effects model (with district fixed effects) to assess changes in the outcome related to changes in the treatment variable. For both sets of analyses, the unit of observation is at the district level since there would be insufficient overlap between the LCMS and MIS at a lower level of observation. Both approaches attempt to control for selection bias, which could explain some or all of the differences in outcomes by degree of malaria control coverage. The authors used these approaches because a priori it is not clear whether it is the *change* in malaria control coverage that matters for micro-economic outcomes or the *level* of coverage. The latter would be identified through the PSM model while the former would be identified in the fixed effects model.

There were advantages and disadvantages to both approaches. The PSM model allowed control for all observable baseline factors that influenced ITN distribution and IRS activities and the outcome variables. Particularly when the NMCC’s approach to selecting the districts that received ITNs and/or IRS was known, the authors attempted to mimic this process via the GPS model. The GPS, which captured the probability that the unit of observation (e.g., district) reached a given level of ITN and IRS coverage as a function of observable baseline characteristics, made the assumption of unconfoundedness: once the authors controlled for the propensity score in the analysis, then malaria control coverage was independent of the outcomes and there were no confounders influencing this relationship [27]. This assumption held if, within each stratum of GPS values, malaria control coverage were independent of baseline characteristics. However, this method also assumed that there were no *unobservable* characteristics that systematically differed by level of coverage and that were related to the outcomes in order to identify causal effects.

The second method used a DD approach that included district fixed effects. This approach estimated the changes over time in the malaria control coverage and its effect on the change in the outcome of interest. The benefit of this approach was that the units of observations did not need to have similar baseline levels of coverage to act as comparisons, since the estimates identified the effect of *change* in coverage rather than the *level* of coverage. In addition, including fixed effects controlled for any time-invariant (but not time-variant) factors at the district level that may have influenced the outcomes. One of the main weaknesses of this approach was that it relied on the ‘equal trends’ assumption; the outcome of interest in areas with high rates of coverage at endline would have changed over time at a similar rate as those with low rates of coverage, in the absence of malaria control scale-up activities. In other words, this approach failed to provide unbiased estimates if micro-economic outcomes would have improved at higher rates in high malaria control coverage areas than in low malaria control coverage areas, even in the absence of these scale-up activities.

The first model predicted the GPS as a function of all available baseline variables in 2006 from the LCMS (see Table 1), as well as malaria parasite infection prevalence from MIS, outpatient malaria cases from HMIS data, and rainfall and elevation measures. The authors also included control variables to account for the NMCC’s targeted approach of districts between 2006 and 2008, classifying districts that were part of the first phase of provinces targeted for malaria control in 2006 (Phase 1 provinces), those targeted in 2007 (Phase 2 provinces) and those targeted in 2008 (Phase 3 provinces) [28–31].^2^ The baseline variables that were selected include key variables that the NMCC used to determine sequencing of malaria targeting; these include malaria parasite infection prevalence and urbanicity.

The first stage predicted the GPS based on these baseline variables and the continuous treatment measure (defined previously). Tests for a normal distribution of the treatment variable given the pre-treatment variables and for the balancing property were conducted [27].^3^ Then the outcome of interest was regressed on the treatment variable, treatment variable squared, GPS, GPS squared, and their interaction. The authors generated a dose–response function, estimated standard errors using bootstrapping, and presented the derivative of the dose–response function. This derivative represents the marginal effect of increasing treatment by a given amount on the outcome of interest, similar to the interpretation of OLS coefficients [32].^4^^,^ 5

In the second model, which used a DD approach with district fixed effects, the data for 2006 and 2010 were pooled, and the same variable definitions as in the GPS model were used for both years. By including district fixed effects, the authors controlled for any unobserved, time-invariant factors at the district level and assess, within the district, the effect of the change in malaria control coverage. The interaction term is malaria control coverage [a proportion (0, 1) variable] interacted with a dummy variable for the endline. The authors used Efron robust standard errors in this analysis to account for variables estimated at the district level.

Reported results are statistically significant at the 95% confidence level or greater (p < 0.05). All analyses were conducted using Stata 13.

### Analytic power

The power calculations were based on data from the 2010 Zambia LCMS. The authors selected monthly household food spending as the primary outcome. Average monthly household spending on food was 406,980 Kwacha (or $81) and it was assumed that the standard deviation is equal to the mean (since raw data were not available at study initiation).^6^ Given a sample size of 20,000 households clustered at the district level (72 districts) and an intra-class correlation of 15% and R-squared of 30%, the authors would be able to detect, with 80% power and a 95% confidence level, an effect size of 89,177 Kwacha or greater; this represented a 22% increase in household food spending per month relative to the average. Other studies have shown substantial variation in the effect of malaria episodes on microeconomic outcomes. The minimum detectable effect size found here was larger than other studies; for example, Dillon et al. [33] found a 7% increase in workers’ earnings from having access to workplace diagnosis and treatment, while Fink et al. [11] found a 15% increase in the average value of agricultural output as a result of access to free ITNs.

### Follow-up simulations regarding sample size and treatment variations

As a follow-up to the analysis, the authors conducted simulations in order to test the extent to which the statistical power would improve using similar data but in settings where: (i) there were more districts than the 72 districts in Zambia; and, (ii) the relationship between malaria control and micro-economic outcomes was either stronger and/or confounding factors could have been more effectively controlled for. For the first assessment, the authors ‘created’ more districts by drawing 1000 datasets with increasing sample sizes (in multiples of the original size of 72 districts, up to 1008 districts). The authors selected with replacement from the original dataset. An additional analysis was to draw 1000 datasets comprised of between 75 and 1000 districts from a random gamma distribution for the outcome and a random beta distribution for the treatment variable. For the second assessment, the authors used the 1000 datasets from the random gamma and beta distributions, and then consecutively increased the amount of unexplained variance in the outcome variables attributable to malaria control from 5 to 50%.

## Results

### Variation in malaria control interventions and micro-economic indicators

The authors first compared baseline and endline estimates of the treatment variable and the outcome variables to describe changes over time and variation across districts. Although the analysis used the district as the unit of analysis, these data are presented by province for ease of interpretation (Table 2); district-level information on coverage can be found in the Table A9 (Additional file 1). Coverage of ITNs and IRS clearly increased in all provinces between 2006 and 2010. On average, ITN and IRS coverage increased from 45 to 73% during this period. Some provinces (e.g., North Western and Northern) saw higher percentage increases in coverage (93 and 170%, respectively), but had lower coverage rates at baseline (40 and 25%) compared to others where coverage rates were already relatively high in 2006 (66% in Western). Table 2 also shows that monthly household food spending, the primary outcome, consistently increased across all provinces during the study period. On average, monthly food spending was 342,264 Kwacha in 2006 and increased by 32% in 2010 to 455,316 Kwacha. The percentage change in total food spending across provinces ranged from 12 to 56%. In contrast, maize production increased only slightly (4.1%) in the country as a whole, with the Northern Province witnessing a 36% increase, while the Copperbelt Province dropped by nearly 22%.

**Table 2:** Baseline and endline variation in malaria control coverage, food spending, and maize production

Province	Coverage of ITNs and/or IRS			Total food spending (000s of 2010 Kwacha)			Maize production (metric tons 000s)		
	2006 (%)	2010 (%)	Change (%)	2006	2010	Change (%)	2006	2010	Change (%)
Central	58	78	35	316	491	56	409	411	0
Copperbelt	54	77	42	483	637	32	206	161	−22
Eastern	42	78	84	252	329	31	436	456	5
Luapula	39	59	51	290	323	12	61	58	−5
Lusaka	34	65	90	528	676	28	92	74	−20
North Western	40	78	93	315	479	52	97	100	3
Northern	25	67	170	269	352	31	198	269	36
Southern	51	75	47	278	379	36	343	402	17
Western	66	78	18	225	283	26	101	100	−1
Average	45	73	62	342	455	33	233	243	4

Values may differ for food spending from the LCMS report due to differing treatment of outliers and possible, unobserved data cleaning or adjustments by the Central Statistical Office. Due to difficulties with the authors’ 2006 agricultural production data, they present the figures for maize production from the LCMS Survey Report 2006 and 2010. Averages are weighted by district population

The correlation coefficients between food spending in 2010 and ITN and/or IRS coverage achieved by 2010 were positive though not large (0.16), suggesting that provinces with higher food spending by 2010 also had higher ITN and/or IRS coverage by 2010. In addition, the authors found that the correlation coefficient between food spending in 2006 and ITN and/or IRS coverage in 2006 was positive (0.16).

### OLS model

When a simple OLS regression of the micro-economic outcomes on *ownership* of ITNs and/or IRS receipt (the treatment variable) was performed, detectable effects were not identified. However, the results were different when the authors examined districts that were part of the first phase of provinces targeted for malaria control in 2006 (Phase 1 provinces), compared to those targeted in 2007 (Phase 2 provinces) and those targeted in 2008 (Phase 3 provinces). When interaction terms between phase of targeting and the treatment variable were included, Phase 2 districts were found to have higher food spending in 2010 compared to those targeted in the first phase (Table 3). In contrast, Phase 1 districts had higher rates of school attendance and higher years of education compared to those targeted in Phase 2.

**Table 3:** Association between malaria control and micro-economic outcomes using OLS with cluster robust standard errors

	Total household spending in 2010 (log)		Total food spending in 2010 (log)		Total food spending in 2010 (log) (no outliers)		Percent of school-age children in school in 2010		Average years of education among school age or older	
	(1)	(2)	(3)	(4)	(5)	(6)	(7)	(8)	(9)	(10)
Per cent of households owning ITN or were sprayed (2010)	0.00	−0.86	0.09	−1.11*	0.01	−0.72	0.07	0.26**	0.78*	2.83***
	(0.28)	(0.58)	(0.31)	(0.64)	(0.22)	(0.47)	(0.06)	(0.11)	(0.46)	(0.72)
Phase 2 provinces		−0.53		−0.77*		−0.53*		0.14*		1.33*
		(0.42)		(0.45)		(0.31)		(0.08)		(0.71)
Phase 3 provinces		0.44		0.07		0.07		0.09		1.72*
		(0.61)		(0.61)		(0.40)		(0.11)		(1.02)
Phase 2 provinces X% of households owning ITN and/or houses sprayed (2010)		1.37*		1.73**		1.08**		−0.25**		−2.34***
		(0.70)		(0.77)		(0.49)		(0.11)		(0.88)
Phase 3 provinces X% of households owning ITN and/or houses sprayed (2010)		0.39		0.88		0.56		−0.12		−2.50*
		(0.79)		(0.81)		(0.52)		(0.13)		(1.29)
Constant	10.47***	12.61***	10.06***	12.45***	11.85***	13.14***	−0.28	−0.68	4.11	1.52
	(2.69)	(2.45)	(2.83)	(2.50)	(2.13)	(1.88)	(0.58)	(0.57)	(5.34)	(4.81)
District level control variables	X	X	X	X	X	X	X	X	X	X
Observations	72	72	72	72	72	72	72	72	72	72
R-squared	0.774	0.836	0.631	0.736	0.739	0.803	0.626	0.685	0.887	0.905
Mean of dependent variable	13.313	13.313	12.896	12.896	12.851	12.851	0.651	0.651	4.724	4.724

Phase 1 is a dummy variable for provinces that were targeted with malaria control scale-up in 2006; these includes Western, North Western, Lusaka, Southern and Luapula. Phase 2 is a dummy for provinces targeted in 2007; these include Northern, Eastern, Southern, and North Western. Phase 3 is a dummy for provinces targeted in 2008; these include Copperbelt, Central, and Lusaka. Phase 1 is the omitted category. The interaction term between Phase 2 and per cent of households owning ITN and/or houses sprayed can be interpreted in two ways: (1) the marginal effect of increasing household ownership of ITNs and/or receipt of IRS by 1% point on micro-economic outcome for districts in Phase 2 provinces, compared to those districts in Phase 2 provinces with 0% household ownership of ITN and/or IRS receipt; or, (2) for a given level of ITN ownership and/or receipt of IRS, the marginal difference in micro-economic outcome between districts in Phase 2 provinces relative to districts in Phase 1 provinces. Each of the specifications includes control variables using 2006 baseline data. They include: number of outpatient malaria visits, malaria parasite infection prevalence for children <5 years old, level of rainfall, elevation, number of children ≤18 years in household, number of children ≤5 years in household, household size, per cent of rural households, percent of household heads who have at least completed primary education, per cent of households with primary employment in different sectors, and the baseline equivalent of the outcome variable

* Significant at 10%; ** significant at 5%; *** significant at 1%

As noted in “Methods” section, the OLS model summarized in Table 3 aimed to describe the relationship between the treatment variable and outcomes using cluster robust standard errors. Because the authors could not distinguish between correlation and causation due to selection bias, additional analysis using Efron robust standard errors was conducted to account for variables being estimated at the district level and to prevent weighing certain observations too heavily in the regression plane. As shown in Table 4, the OLS results were no longer statistically significant after making this correction. The only significant finding is for years of schooling among districts in Phase 1: the authors continue to see that those districts have higher schooling attainment by 2010.

**Table 4:** Association between malaria control and micro-economic outcomes using OLS with Efron standard errors

	Total household spending in 2010 (log)	Total food spending in 2010 (log)	Total food spending in 2010 (log) (no outliers)	Percent of school-age children in school in 2010	Average years of education among school age or older
	(1)	(2)	(3)	(4)	(5)
Per cent of households owning ITN or were sprayed (2010)	−0.86	−1.11	−0.72	0.26	2.83**
	(1.00)	(1.11)	(0.83)	(0.16)	(1.13)
Phase 2 provinces	−0.53	−0.77	−0.53	0.14	1.33
	(0.71)	(0.76)	(0.55)	(0.11)	(0.98)
Phase 3 provinces	0.44	0.07	0.07	0.09	1.72
	(1.01)	(1.03)	(0.66)	(0.15)	(1.62)
Phase 2 provinces X% of households owning ITN and/or houses sprayed (2010)	1.37	1.73	1.08	−0.25	−2.34*
	(1.18)	(1.29)	(0.85)	(0.16)	(1.27)
Phase 3 provinces X% of households owning ITN and/or houses sprayed (2010)	0.39	0.88	0.56	−0.12	−2.50
	(1.32)	(1.38)	(0.88)	(0.19)	(2.08)
Constant	12.61***	12.45***	13.14***	−0.68	1.52
	(3.81)	(3.88)	(2.91)	(0.81)	(7.09)
District level control variables	X	X	X	X	X
Observations	72	72	72	72	72
R-squared	0.836	0.736	0.803	0.685	0.905
Mean of dependent variable	13.313	12.896	12.851	0.651	4.724

Phase 1 is a dummy variable for provinces that were targeted with malaria control scale-up in 2006; these includes Western, North Western, Lusaka, Southern and Luapula. Phase 2 is a dummy for provinces targeted in 2007; these include Northern, Eastern, Southern, and North Western. Phase 3 is a dummy for provinces targeted in 2008; these include Copperbelt, Central, and Lusaka. Phase 1 is the omitted category. The interaction term between Phase 2 and percent of households owning ITN and/or houses sprayed can be interpreted in two ways: (1) the marginal effect of increasing household ownership of ITNs and/or receipt of IRS by 1% point on micro-economic outcome for districts in Phase 2 provinces, compared to those districts in Phase 2 provinces with 0% household ownership of ITN and/or IRS receipt; or (2) for a given level of ITN ownership and/or receipt of IRS, the marginal difference in micro-economic outcome between districts in phase 2 provinces relative to districts in Phase 1 provinces. Each of the specifications includes control variables using 2006 baseline data. They include: number of outpatient malaria visits, malaria parasite infection prevalence for children <5 years old, level of rainfall, elevation, number of children ≤18 years in household, number of children ≤5 years in household, household size, per cent of rural households, per cent of household heads who have at least completed primary education, per cent of households with primary employment in different sectors, and the baseline equivalent of the outcome variable. The authors used Efron cluster robust standard errors to account for the fact that the outcomes measures are drawn from a sample not intended to be representative at the district level

* Significant at 10%; ** significant at 5%; *** significant at 1%

### GPS model

**Fig. 2:**
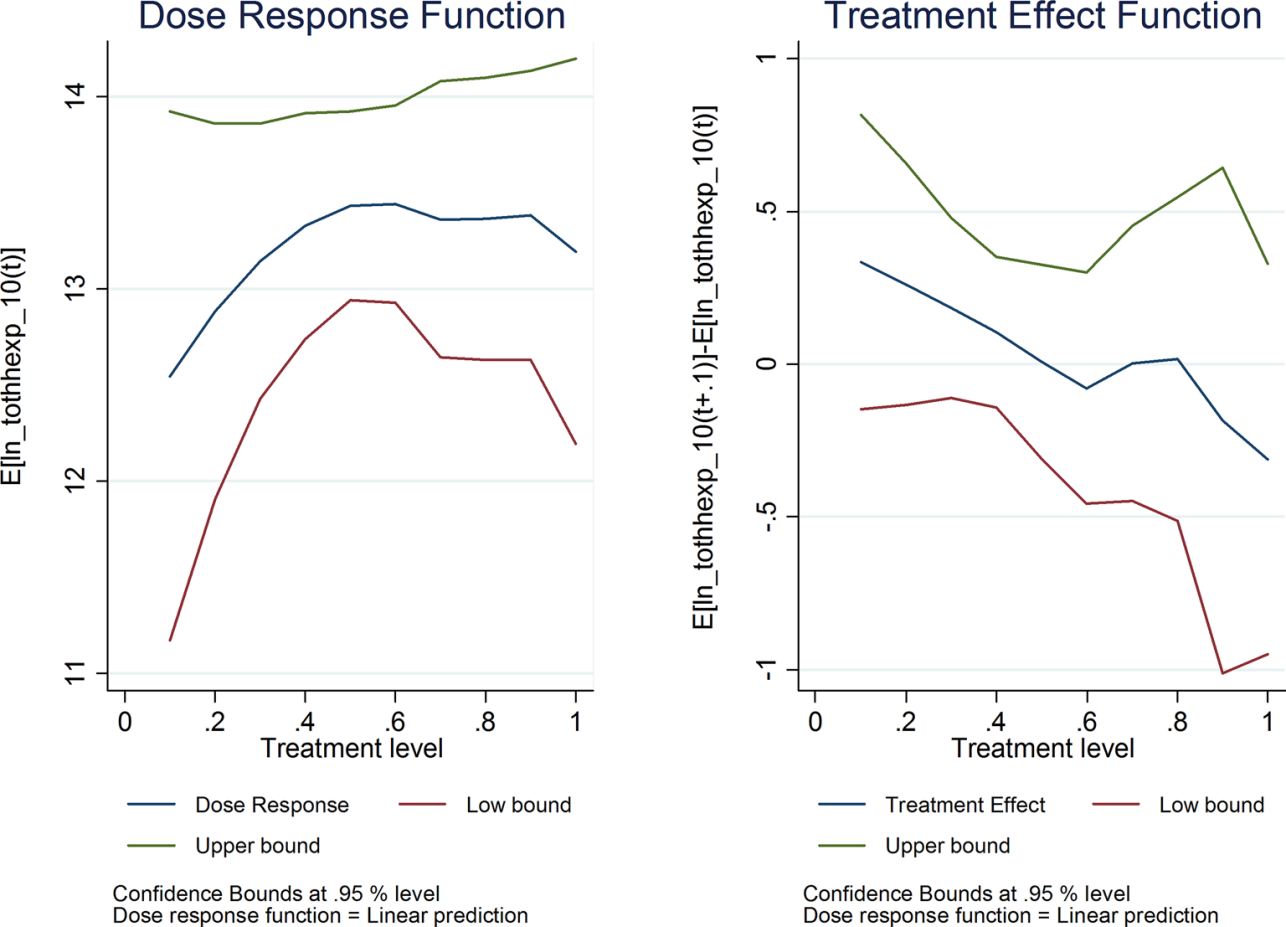
ITN/IRS ownership by 2010 and ln (total household expenditures) in 2010

The first set of results from the GPS model assessed the relationship between *ownership* of ITNs and/or receipt of IRS and the micro-economic outcomes of interest. Two graphs for each outcome are presented (Fig. 2). The first shows the dose–response function, relating each level of the treatment variable (i.e., ownership) to the level of the outcome. The second is the treatment effect function, showing the marginal effect on the outcome of increasing treatment by 10% points (i.e., ownership of ITNs and/or receipt of IRS increases from 60 to 70%); these parameters can be interpreted similarly to OLS regression coefficients. The x-axis denotes the treatment variable, specifically the percentage of population owning ITNs and/or whose dwelling was subjected to IRS.

Overall, the authors found no statistically significant relationships between ITN *ownership* and/or IRS receipt and either primary (total household spending on food) or secondary (e.g., educational attainment, agricultural production, wage labour, medical spending, education spending, and borrowing) outcomes. For all outcomes, the confidence intervals were large enough that a positive, negative or null relationship could not be ruled out. Next, the authors discuss the estimated coefficient related to increasing treatment from 60 to 70%, since mean ITN ownership and/or IRS receipt is 73% by 2010 (see Additional file 1 for dose–response coefficients). While this coefficient was expected to be positive, it is not positive in all cases. The estimated coefficient’s sign also varies depending on the level of treatment.

**Fig. 3:**
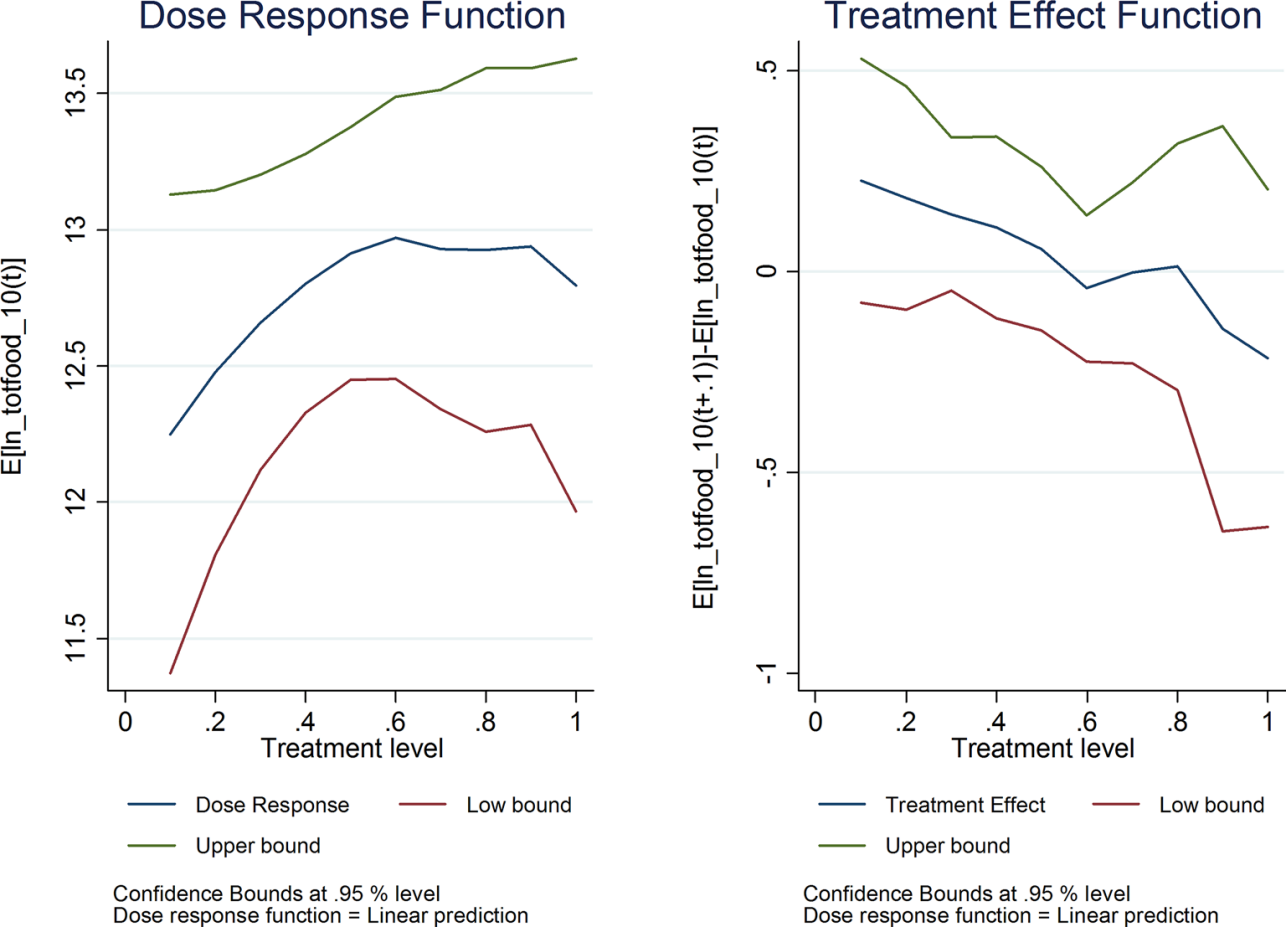
ITN/IRS ownership by 2010 and ln (household food spending) in 2010

**Fig. 4:**
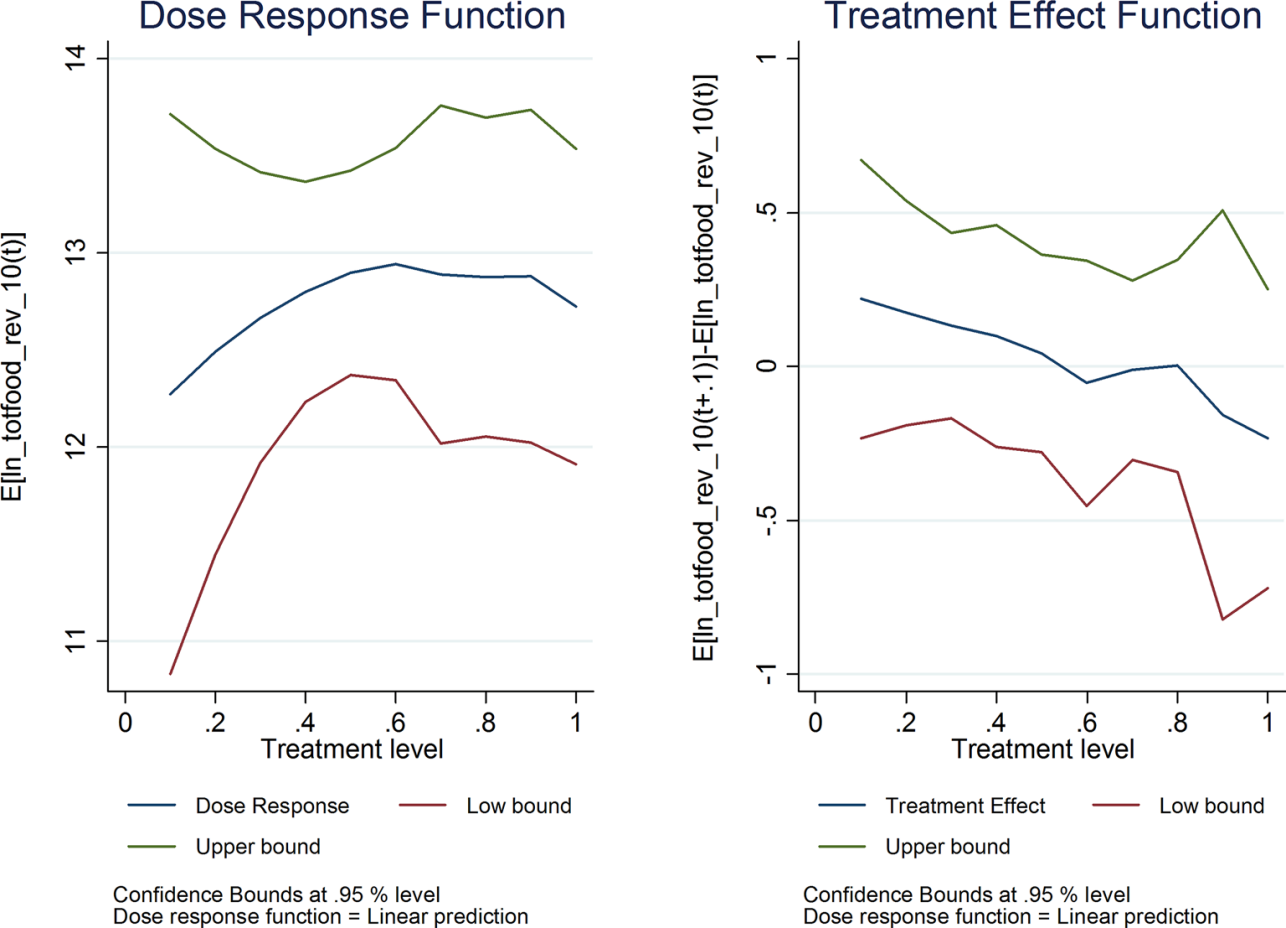
ITN/IRS ownership by 2010 and ln (household food spending, outliers removed) in 2010

For spending on food, statistically significant associations were not found. The estimated coefficient is 4%; the confidence intervals indicate that the authors can rule out a positive association greater than 14% when ITN ownership and/or IRS receipt increases from 60 to 70% (Fig. 3). When household level observations that appear to be outliers are removed, the largest effect that can be ruled out is a 34% increase (Fig. 4).

**Fig. 5:**
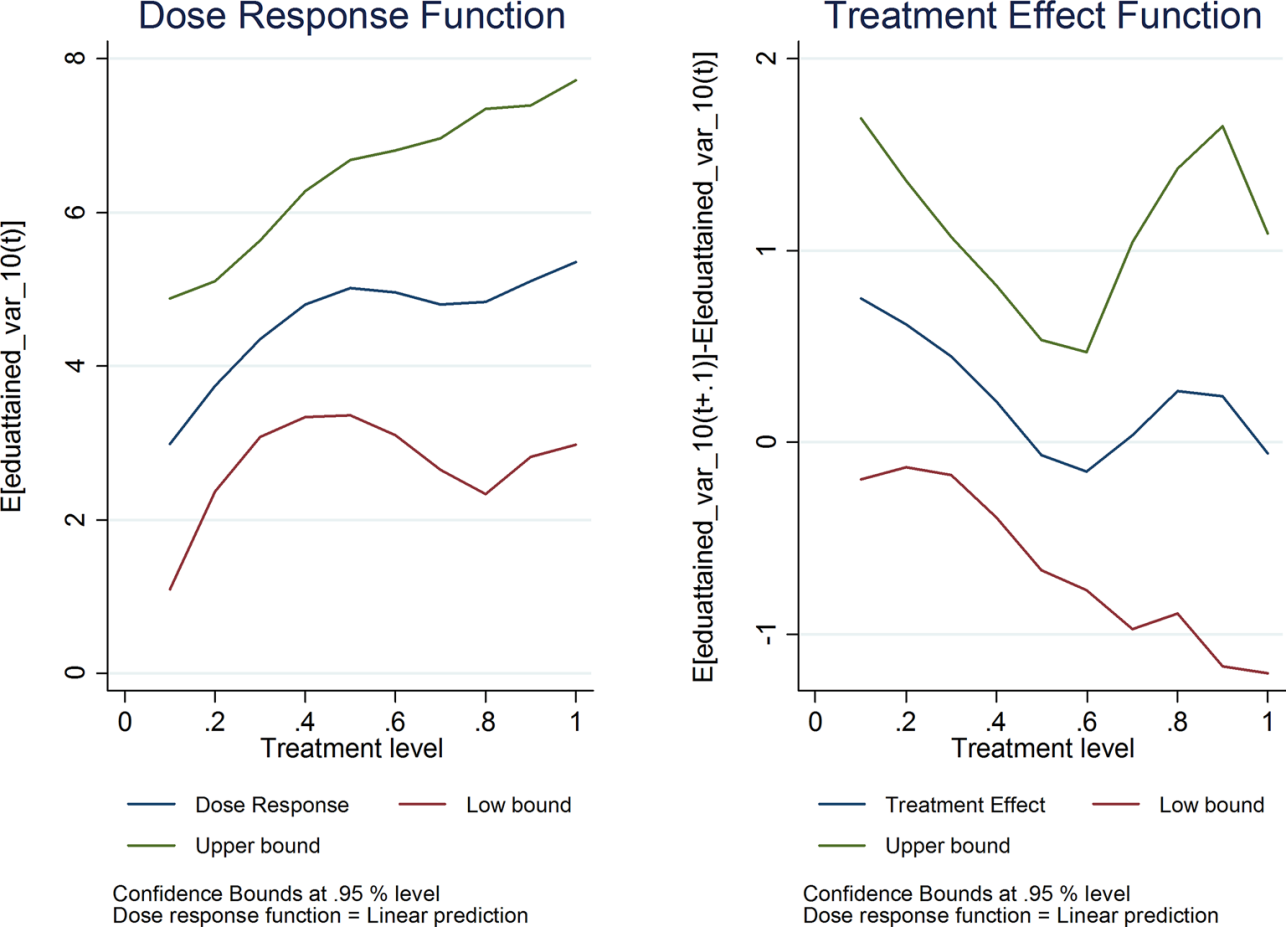
ITN/IRS ownership by 2010 and years of schooling in 2010. Measured for respondents school-aged or older in 2010

Similarly, for education, statistically significant results were not identified. The confidence interval rules out an effect larger than half a year of schooling, meaning that if malaria control scale-up has a positive effect on years of schooling, the effect will be under a half a year (Fig. 5).

When assessing agricultural production in the last year, the authors found no statistically significant correlations between the treatment variable and the production of maize, nuts, or potato. The upper bound estimate for maize production represents a 57% increase in production (Table A8a in Additional file 1). The magnitude of this confidence interval is very large.

Additional results (available in Additional file 2) display results on the relationship between the *use* of ITNs and/or receipt of IRS and microeconomic outcomes. The results are similar to those for the ownership treatment measure. So too are results for certain secondary microeconomic outcomes: wage labour, medical spending, education spending, and borrowing (in Additional file 3).

### Fixed effects model

As in the GPS model, the fixed effects models did not find statistically significant relationships between malaria control scale-up and the primary or secondary micro-economic outcomes (Table 5). The key coefficient to interpret is the interaction term between a dummy for the endline period and the malaria treatment variable, which is usage/ownership of ITNs and/or the IRS receipt. Across the Tables, this coefficient was found to be statistically insignificant. For example, the coefficient for this interaction term in the model of maize production and ITN usage and/or IRS receipt indicates that a 10% increase in coverage would result in an improvement in total food spending of 0.3% at endline. However, the confidence interval around this estimate was wide (−55 to 139%). This was also the case with the other fixed effects models, such that a positive, negative, or null change in maize production could not be ruled out.

**Table 5:** Association between malaria control and micro-economic outcomes—fixed effects analysis

	Total household spending (log) (no outliers)	Total food spending (log) (no outliers)	Percent of school-age children in school	Average years of education among school age or older
	(1)	(2)	(3)	(4)
Endline dummy	0.00	0.23	−0.05	−0.77
	(0.34)	(0.29)	(0.10)	(0.87)
Per cent of households owning ITN and/or houses sprayed	0.08	−0.06	0.02	−0.28
	(0.41)	(0.34)	(0.09)	(0.63)
Interaction between endline dummy and per cent of households owning ITN and/or houses sprayed	0.08	0.03	0.13	1.01
	(0.48)	(0.42)	(0.16)	(1.38)
Constant	13.34***	12.81***	0.16	4.79
	(2.52)	(1.94)	(0.67)	(5.13)
District fixed effects	X	X	X	X
District level control variables	X	X	X	X
Observations	144	144	144	144
R-squared	0.907	0.890	0.803	0.949
Mean of dependent variable	13.245	12.715	0.644	4.655

See Table 4 for an explanation for all the variables in this analysis. From that list of variables, the authors excluded district level controls variables that are time-invariant including elevation and dummy variables for provinces that were targeted with malaria control scale. The authors used Efron cluster robust standard errors to account for the fact that the outcomes measures are drawn from a sample not intended to be representative at the district level

* Significant at 10%; ** significant at 5%; *** significant at 1%

### Simulations

Based on the simulations, the authors found that even a substantial increase in the sample size would not be sufficient to reliably detect an effect on food spending using a GPS model, given the variation in this outcome and malaria control efforts in the data for Zambia from 2006 to 2010. Even with a sample size of 1008 districts, the GPS model only detected an association between malaria control and food spending in 21% of the iterations. Results based on simulated data from the estimated distributions showed even lower power. Even with the GPS model simulated to explain 50% of food spending, the model only detected an association in 5–6% of the iterations.

## Discussion

In this study, the authors used a quasi-experimental design and drew upon commonly available secondary data on malaria coverage and micro-economic outcomes to provide robust evidence of the relationship between malaria control coverage and micro-economic outcomes at national level in Zambia, a country that has scaled-up malaria control efforts and reduced the incidence of malaria. Reliable, country-specific evidence would contribute to a better understanding of the return on investment from scaling-up malaria control efforts and achieving the objective of malaria elimination, especially as governments and donors face competing demands on limited resources.

The substantial scale-up of malaria control efforts in Zambia from 2006 to 2010 combined with the availability of data coinciding with this period provided a unique opportunity to use secondary data and a rigorous analytical approach. The authors observed improvements in coverage of malaria control efforts in parallel with improvements in micro-economic outcomes, such as food spending, during the study period. The authors also found that food spending in 2006 was positively correlated with ITN and/or IRS coverage in 2006. These positive correlations suggest that either higher ITN and/or IRS coverage achieved by 2010 caused higher food spending by 2010 (i.e., a positive micro-economic impact), or that areas that tend to have higher malaria control coverage tend to be areas with higher food spending to begin with. The quasi-experimental approaches attempted to control for this second association, which represents a selection effect and confounds the relationship between malaria control scale-up and micro-economic outcomes.

When exploratory OLS analyses of the correlation between the scale-up over time of malaria control efforts and micro-economic outcomes was performed, the authors found that districts targeted earlier for malaria control scale-up (which are called Phase 1 districts in this study) had higher rates of school attendance and more years of education, but lower food spending by 2010 than those targeted for later efforts. One interpretation is that Phase 1 districts, by virtue of being targeted earlier and over a longer period, had better economic outcomes by 2010, as proxied by educational attainment. However, the lower food spending by 2010 suggests that the authors may also be capturing some possible selection bias; Phase 1 districts may have had worse outcomes to begin with, such as lower food spending, and therefore were targeted first. While these exploratory OLS results (along with the initial correlations identified) do not provide conclusive evidence suggesting that malaria control efforts were targeted to specific areas, they further highlight the need for quasi-experimental approaches to generate unconfounded results that allow for causal inference related to the impact of malaria control efforts.

For the GPS model, the authors failed to identify statistically significant relationships between ITN ownership and/or IRS receipt and the primary (total household spending on food) or secondary micro-economic outcomes. For food spending, the confidence intervals were such that positive associations between malaria control coverage and micro-economic outcomes greater than 14 or 34% could be rule out, depending on specification. To put this into context, for households with average food spending among the lowest quintile for food spending in 2010, an increase of 14 or 34% in food spending would not move them out of the lowest quintile; indeed, it would require a 49% increase in average food spending of those in the lowest quintile to move them up to the next quintile.

For the secondary outcome, educational attainment, the confidence interval around the GPS coefficient rules out an effect greater than half a year of schooling. The authors can compare this upper bound with results from other studies focusing on educational attainment. A retrospective study in Uganda of a malaria eradication programme showed that educational attainment increased by a half a year for both women and men [14]. Another retrospective study found that national malaria eradication campaigns in Sri Lanka and Paraguay increased completed schooling by approximately 0.1 years [17]. These studies suggest that the bounded estimate is within the range of other studies, if there is in fact a positive impact of malaria control coverage on educational attainment. That said, other studies have also found no effect on educational attainment [15, 18], and the authors similarly cannot rule out that there was no impact on educational attainment.

For agricultural production, the GPS results ruled out an effect greater than a 57% increase in maize production. By comparison, Fink et al. identified a 15% increase in agricultural output as a result of providing free bed nets. The results clearly include this effect but are too large to make any meaningful conclusions. In addition, there still remains evidence of imbalance in certain analyses, specifically for agricultural production, even after controlling for the GPS.

As with the GPS model, the fixed effects model did not find statistically significant relationships between malaria control scale-up and either primary or secondary micro-economic outcomes. Confidence intervals were similarly wide, making it impossible to rule out positive, negative or null changes in these outcomes.

One factor that may explain the absence of detectable effects in this specific example is the resurgence of malaria in certain provinces of Zambia over this time period. Malaria parasite infection prevalence declined rapidly from 2006 to 2008 in nearly all provinces of Zambia, but then increased again in six of nine provinces by 2010, according to MIS data [34]. These trends indicate that malaria control programmes may not have maintained as high levels of coverage in the second half of the study period, possibly due in part to reduced funding for commodities and delays with procurement and implementation, which led to ITNs not being delivered [35]. Despite summary statistics showing large increases in coverage during this period, these figures may mask lower changes in prevalence if malaria control scale-up was not effectively implemented in practice. Additionally, reported use of ITNs used in the treatment measure may well be higher than actual use due to desirability bias, which would attenuate the relationship between the malaria control and micro-economic outcomes.

While the results are inconclusive, the approach taken in this study can yield lessons for key stakeholders, including donors, governments, and other researchers interested in investigating the link between malaria control scale-up and micro-economic outcomes. These lessons illuminate the challenges encountered in using secondary data, including selection effects, limited sample size, data limitations, and other potential confounders, and suggested approaches for addressing them.

Using low-cost, reliable, representative and readily available data offers clear benefits in answering policy-relevant questions using rigorous analytic approaches. Specifically for researchers and stakeholders conducting malaria-related research, there is ready access to standardized malaria indicator data on both ITN distribution and IRS available for 30 countries in Africa through MIS. Many countries have multiple years of data from the MIS, allowing for longitudinal analyses and nationally representative findings. In addition, programmatic data on net distribution and spraying may also be available, along with micro-economic data from integrated living standards surveys (like the LCMS) on outcomes such as household consumption, agricultural production and education. Use of these existing, secondary data, coupled with a quasi-experimental approach, provides a resource-friendly alternative to stand-alone data collection efforts. In addition, if there is sufficient variation in the intervention and it is possible to identify similar comparison groups, a quasi-experimental approach can provide convincing causal evidence, in the absence of a randomized experiment.

The first lesson from the study relates to the challenges of using nationally representative data, which may not be representative at lower geographic units, such as sub-district. The authors discovered that conducting the analysis at the district level significantly reduced the study’s statistical power. There were 72 observations, the total number of districts in Zambia in 2010, for the analysis. The power calculations showed that this model could detect a 22% increase in food spending, yet the upper bound of the confidence intervals from the GPS results showed that the authors were able to reject an effect greater than 14–34%, depending on specification. The confidence intervals for agricultural production are even larger. Other studies, such as Fink et al. [11], identified a 15% increase in agricultural production which suggests that this study was under-powered to detect these positive impacts. One potential solution would be to conduct the analysis at a lower geographical level, which would increase the statistical power but may introduce other challenges. For example, analysing these data at a lower geographic unit runs the risk of environmental bias, meaning that the randomly selected sampling unit within a given area may have better or worse outcomes by chance than the average in the area.

Moreover, while there are clear benefits from using low-cost, readily available, national-level, secondary data, a key consideration relates to the feasible unit of analysis that can be used. This is especially important when multiple sources of data must be merged into a single analytic dataset, and when some of the sources are only available at the district level or higher. For example, some data, such as NMCC data on net distribution, was only available at the district level. The sample sizes required can be large, as the simulations demonstrated for Zambia over the period of study. Smaller sample sizes would be suitable if the predicted effect is assumed to be large; in this case, only limited evidence of effect size was available at the time of the study design.

The second lesson relates to whether using a quasi-experimental design drawing on secondary data can sufficiently and convincingly eliminate potential selection effects. The OLS regressions found evidence of potential selection bias, meaning that provinces which were targeted earlier for malaria control scale-up had lower food spending by 2010 but also ended up having better educational outcomes. These contradictory findings point to potential selection effects (i.e., provinces targeted earlier were those with poorer outcomes such as lower food spending) combined with the potential impacts of malaria control interventions (i.e., provinces targeted earlier had better educational outcomes after scale-up). These potential selection effects are a prime reason for selecting quasi-experimental designs. In the GPS analyses, despite mitigating selection effects by controlling for baseline characteristics used to generate the propensity score, the authors still found evidence of imbalance (meaning that there still remain unobservable factors that differentiate districts). Factors, such as political considerations that may have influenced district selection for scale-up efforts as well as ease of implementation of malaria control efforts, are challenging to control for analytically and are not available through these secondary data sources. By complementing the GPS approach with the fixed effects model, the authors attempted to control for time-invariant characteristics at the district. Yet, the large confidence intervals highlight that this study still had limited statistical power. The simulation findings indicate that the GPS model is not a viable choice for examining the relationship between malaria control and food expenditures in Zambia.

The third lesson highlights the potential benefits of using primary versus secondary data, which can influence factors such as the authors’ ability to control for selection bias as discussed. With primary data collection, researchers have more control over the data collected, both at baseline and follow-up. There would no longer be a concern with merging data sources at different levels of disaggregation or representative populations. In the case of malaria, it would also be more useful to have data with a very short recall period since the micro-economic impact of a malaria episode may not necessarily be identified through measures aggregated across a year. For example, data on days of work lost, school days missed, food spending, and household financial coping strategies in the last month would be much more useful in identifying an impact on household’s economic well-being. The available schooling measure in the LCMS (whether a child is attending school in general) is a crude approximation that is not likely to capture the effect of a malaria episode on days of school missed. While developing survey instruments that collect the necessary variables would provide the exact measures needed, primary data collection is much more costly than relying on secondary datasets. These costs need to be weighed against the reliability of estimates that can be produced using existing secondary data.

One recommendation is to add additional survey questions to large-scale household surveys, such as the MIS, the LCMS, the Living Standards Measurement Studies, and the Demographic Health Surveys. For example, the LCMS in Zambia could add a small set of questions on malaria coverage and therefore permit an examination of the research question of this study at the household level. Alternatively, the MIS could incorporate micro-economic outcomes into its survey instrument. The challenge though is that certain variables such as food consumption and agricultural production require large modules to collect reliable estimates. High-quality implementation data would also be helpful. In this study, the authors intended to use data from the NMCC on ITN distribution by district to look at the impact of donor and national government’s malaria control efforts. However, when these data were correlated with the MIS ITN ownership data, the authors found a very low correlation and determined that more reliable estimates would come from the MIS data. Nonetheless, implementation data from the NMCC, especially if available at a lower geographical area, would be a low-cost, alternative data source for this type of study.

The fourth lesson relates to the selection of research methodology. Where there are relative merits of using a retrospective, quasi-experimental approach compared to other research designs, there are accompanying challenges as well. One of the reasons the authors chose a retrospective, quasi-experimental design was the potential challenge of randomizing access to malaria control interventions. One important ethical concern of donors and implementers is the prospect of withholding effective malaria control measures for an extended time period. However, randomized experiments seek to overcome this limitation in various ways, such as varying price for nets. There are clear benefits from using a randomized experiment, in terms of causal attribution. Evaluators can conclusively attribute any microeconomic effects to the malaria control interventions, as in Fink et al. [11] where the impact on farmer productivity can be directly attributed to having access to free bed nets. However, other factors, such as programmatic feasibility of varying access to an intervention, particularly over longer periods of time, may make a randomized experiment less feasible or desirable. In this case, using retrospective, commonly available data over a longer period of time allowed a national scope, over a multi-year period, which may yield different results than smaller scale randomized experiments implemented in a small geographic area.

## Conclusions

This study complements existing literature by using a national scope, a multi-year research period, and commonly available survey data in the context of Zambia. The research question: whether malaria control scale-up is associated with improved microeconomic outcomes, is an important one for other African countries, donors and implementers working to combat malaria. However, there are important challenges to address in utilizing retrospective, existing data sources on malaria and micro-economic outcomes, most notably regarding the level at which data on both the outcomes and malaria control measures can be disaggregated. Given these revealed limitations in the collated dataset used and statistical power, the GPS and fixed effects models did not detect statistically significant associations between malaria control and microeconomic outcomes. The authors were able to bound the possible effect sizes, however, such that a positive impact of malaria control on micro-economic outcomes found in recently published studies cannot be ruled out. Future research should carefully assess the possible sample size, expected effect estimates, and disaggregation of the existing, commonly available data. Similarly, more work is needed to define methods and conditions under which use of these data has sufficient precision to isolate an association between malaria control and micro-economic outcomes. In the meantime, unless there are suitable data for a very large number of sub-district clusters available, the authors recommend a prospective study using household level data integrating malaria control coverage and micro-economic outcomes. Alternatively, large surveys like the LCMS could incorporate malaria coverage indicators or, with greater complexity, the MIS could incorporate micro-economic outcomes such as household expenditures and days of normal activity lost into data collection.

## Additional files

**Additional file 1.** Additional tables that share additional results of the generalized propensity score matching analysis, as well as a table covering malaria control coverage at the district level.

**Additional file 2.** Additional figures presenting results with ownership treatment variable.

**Additional file 3.** Additional figures presenting results for use treatment measure and secondary microeconomic outcomes: wage labour, medical spending, education spending, and borrowing.

## Abbreviations

CGIAR-CSI: Consortium for Spatial Information
DD: difference in differences
GPS: generalized propensity score
HMIS: health management information system
IRS: indoor residual spraying
ITN: insecticide-treated net
LLIN: long-lasting insecticide-treated net
LCMS: Living Conditions Monitoring Survey
MIS: Malaria Indicator Survey
NASA: National Aeronautics and Space Administration
NMCC: National Malaria Control Centre
OLS: ordinary least squares
PSM: propensity score matching
RCT: randomized control trial
SRTM: Shuttle Radar Topography Mission
WHO: World Health Organization
USAID: United States Agency for International Development

## Footnotes

1 ‘ITNs’ in both datasets include both ITNs and LLINs; for simplicity the authors refer to both as ITNs. The MIS includes non-factory nets retreated within the last year.

2 Based on data from NMCC action plans, the authors identified the sequencing of malaria control scale-up by provinces. Phase 1 provinces were targeted first in 2006 (Western, North Western, Lusaka, Southern and Luapula), Phase 2 provinces were targeted in 2007 (Northern, Eastern, Southern, and North Western), and Phase 3 provinces were targeted in 2008 (Copperbelt, Central, and Lusaka).

3 The balancing property tests for whether the conditional mean of the pre-treatment variables given the GPS is not different between units within a certain treatment interval and those in other treatment intervals.

4 Bia and Mattei [32] developed a user-written programme, which estimates the GPS score and produces the dose–response function. The code was developed for Stata and specifically follows the steps laid out in Hirano and Imbens [27] to generate GPS.

5 In the main specification, the authors do not use any transformation of the treatment variable. Instead, they use three cut points to divide the continuous treatment variable, evaluate the GPS score at the midpoint of each treatment variable, and use two blocks of the GPS score within each of the three cut points.

6 Since the authors did not have access to the raw data at the time of the power calculations, they used this assumption since the variance of the outcome of interest was not reported in the report.
