# Supplementary material for: Exploring the use of routinely-available, retrospective data to study the association between malaria control scale-up and micro-economic outcomes in Zambia
Source: Malar J. 2017 Jan 4;16:15. doi: 10.1186/s12936-016-1665-z (PMC5209918; doi:10.1186/s12936-016-1665-z)
Supplement: Supplementary file 1 — Additional file 1. Additional tables that share additional results of the generalized propensity score matching analysis, as well as a table covering malaria control coverage at the district level. [file 12936_2016_1665_MOESM1_ESM.docx]

**Additional tables presenting results of the generalized propensity score matching analysis and malaria control coverage by district**

| Table A1: Ownership of ITNs and/or receipt of IRS by 2010 and total household spending (log) in 2010 | | | | | |
| --- | --- | --- | --- | --- | --- |
| Treatment level | Treatment level + 10 percentage points | Treatment Effect | Standard Error | Lower bound | Upper bound |
| 10% | 20% | 0.33 | 0.25 | -0.147 | 0.816 |
| 20% | 30% | 0.26 | 0.20 | -0.134 | 0.657 |
| 30% | 40% | 0.18 | 0.15 | -0.110 | 0.479 |
| 40% | 50% | 0.10 | 0.13 | -0.143 | 0.352 |
| 50% | 60% | 0.01 | 0.16 | -0.308 | 0.326 |
| 60% | 70% | -0.08 | 0.19 | -0.457 | 0.299 |
| 70% | 80% | 0.00 | 0.23 | -0.447 | 0.453 |
| 80% | 90% | 0.02 | 0.27 | -0.512 | 0.546 |
| 90% | 100% | -0.19 | 0.42 | -1.014 | 0.643 |
| 100% | 110% | -0.31 | 0.33 | -0.951 | 0.329 |
|  |  |  |  |  |  |
| Table A2a: Ownership of ITNs and/or receipt of IRS by 2010 and total food spending (log) in 2010 | | | | | |
| Treatment level | Treatment level + 10 percentage points | Treatment Effect | Standard Error | Lower bound | Upper bound |
| 10% | 20% | 0.23 | 0.15 | -0.08 | 0.53 |
| 20% | 30% | 0.18 | 0.14 | -0.10 | 0.46 |
| 30% | 40% | 0.14 | 0.10 | -0.05 | 0.33 |
| 40% | 50% | 0.11 | 0.12 | -0.12 | 0.34 |
| 50% | 60% | 0.06 | 0.10 | -0.15 | 0.26 |
| 60% | 70% | -0.04 | 0.09 | -0.22 | 0.14 |
| 70% | 80% | 0.00 | 0.11 | -0.23 | 0.22 |
| 80% | 90% | 0.01 | 0.16 | -0.29 | 0.32 |
| 90% | 100% | -0.14 | 0.26 | -0.65 | 0.36 |
| 100% | 110% | -0.22 | 0.21 | -0.64 | 0.20 |

| Table A2b: Ownership of ITNs and/or receipt of IRS by 2010 and total food spending (log) (removing outliers) in 2010 | | | | | |
| --- | --- | --- | --- | --- | --- |
| Treatment level | Treatment level + 10 percentage points | Treatment Effect | Standard Error | Lower bound | Upper bound |
| 10% | 20% | 0.22 | 0.23 | -0.23 | 0.67 |
| 20% | 30% | 0.18 | 0.19 | -0.19 | 0.54 |
| 30% | 40% | 0.13 | 0.15 | -0.17 | 0.43 |
| 40% | 50% | 0.10 | 0.18 | -0.26 | 0.46 |
| 50% | 60% | 0.04 | 0.16 | -0.28 | 0.36 |
| 60% | 70% | -0.05 | 0.20 | -0.45 | 0.34 |
| 70% | 80% | -0.01 | 0.15 | -0.30 | 0.28 |
| 80% | 90% | 0.00 | 0.18 | -0.34 | 0.35 |
| 90% | 100% | -0.16 | 0.34 | -0.82 | 0.51 |
| 100% | 110% | -0.23 | 0.25 | -0.72 | 0.25 |
|  |  |  |  |  |  |
| Table A3a: Ownership of ITNs and/or receipt of IRS by 2010 and probability of being in school (for school-aged children) in 2010 | | | | | |
| Treatment level | Treatment level + 10 percentage points | Treatment Effect | Standard Error | Lower bound | Upper bound |
| 10% | 20% | 0.00 | 0.04 | -0.07 | 0.08 |
| 20% | 30% | 0.00 | 0.03 | -0.05 | 0.06 |
| 30% | 40% | 0.00 | 0.02 | -0.04 | 0.04 |
| 40% | 50% | 0.00 | 0.01 | -0.03 | 0.02 |
| 50% | 60% | 0.00 | 0.02 | -0.05 | 0.04 |
| 60% | 70% | 0.00 | 0.02 | -0.05 | 0.04 |
| 70% | 80% | 0.01 | 0.02 | -0.04 | 0.05 |
| 80% | 90% | 0.01 | 0.02 | -0.04 | 0.06 |
| 90% | 100% | 0.01 | 0.05 | -0.08 | 0.10 |
| 100% | 110% | 0.01 | 0.05 | -0.10 | 0.12 |
|  |  |  |  |  |  |
| Table A3b: Ownership of ITNs and/or receipt of IRS by 2010 and years of schooling in 2010 (for school aged respondents or older) in 2010 | | | | | |
| Treatment level | Treatment level + 10 percentage points | Treatment Effect | Standard Error | Lower bound | Upper bound |
| 10% | 20% | 0.75 | 0.48 | -0.19 | 1.69 |
| 20% | 30% | 0.62 | 0.38 | -0.13 | 1.36 |
| 30% | 40% | 0.45 | 0.32 | -0.17 | 1.07 |
| 40% | 50% | 0.21 | 0.31 | -0.39 | 0.82 |
| 50% | 60% | -0.07 | 0.31 | -0.67 | 0.53 |
| 60% | 70% | -0.15 | 0.32 | -0.77 | 0.47 |
| 70% | 80% | 0.04 | 0.51 | -0.97 | 1.04 |
| 80% | 90% | 0.27 | 0.59 | -0.89 | 1.43 |
| 90% | 100% | 0.24 | 0.72 | -1.17 | 1.65 |
| 100% | 110% | -0.06 | 0.59 | -1.20 | 1.09 |

| Table A4a: Ownership of ITNs and/or IRS by 2008 and total maize production (kg) in 2009 | | | | | |
| --- | --- | --- | --- | --- | --- |
| Treatment level | Treatment level + 10 percentage points | Treatment Effect | Standard Error | Lower bound | Upper bound |
| 10% | 20% | -10.3 | 161.7 | -327.4 | 306.7 |
| 20% | 30% | 20.2 | 134.0 | -242.3 | 282.8 |
| 30% | 40% | 51.6 | 169.7 | -280.9 | 384.1 |
| 40% | 50% | 42.6 | 148.1 | -247.7 | 332.9 |
| 50% | 60% | 0.4 | 243.9 | -477.6 | 478.5 |
| 60% | 70% | 8.4 | 200.7 | -385.0 | 401.7 |
| 70% | 80% | 26.4 | 221.6 | -407.9 | 460.7 |
| 80% | 90% | 108.1 | 234.1 | -350.8 | 567.0 |
| 90% | 100% | 365.9 | 251.3 | -126.6 | 858.5 |
| 100% | 110% | 511.2 | 294.5 | -66.1 | 1088.5 |
|  |  |  |  |  |  |
| Table A4b: Ownership of ITNs and/or IRS by 2008 and total potato production (kg) in 2009 | | | | | |
| Treatment level | Treatment level + 10 percentage points | Treatment Effect | Standard Error | Lower bound | Upper bound |
| 10% | 20% | 11.9 | 19.1 | -25.5 | 49.4 |
| 20% | 30% | 6.6 | 15.2 | -23.2 | 36.4 |
| 30% | 40% | 2.1 | 12.2 | -21.8 | 25.9 |
| 40% | 50% | -0.6 | 10.5 | -21.1 | 19.9 |
| 50% | 60% | -2.9 | 7.4 | -17.3 | 11.6 |
| 60% | 70% | 1.4 | 7.0 | -12.4 | 15.2 |
| 70% | 80% | 2.8 | 10.7 | -18.2 | 23.9 |
| 80% | 90% | -7.0 | 11.3 | -29.1 | 15.0 |
| 90% | 100% | -13.2 | 16.4 | -45.4 | 18.9 |
| 100% | 110% | -14.9 | 16.5 | -47.2 | 17.4 |
|  |  |  |  |  |  |
| Table A4c: Ownership of ITNs and/or IRS by 2008 and total nuts production (kg) in 2009 | | | | | |
| Treatment level | Treatment level + 10 percentage points | Treatment Effect | Standard Error | Lower bound | Upper bound |
| 10% | 20% | 13.0 | 21.3 | -28.8 | 54.7 |
| 20% | 30% | 7.3 | 19.4 | -30.8 | 45.4 |
| 30% | 40% | 1.9 | 15.9 | -29.3 | 33.1 |
| 40% | 50% | 0.0 | 13.2 | -25.9 | 25.8 |
| 50% | 60% | 1.8 | 10.5 | -18.8 | 22.3 |
| 60% | 70% | 1.2 | 10.0 | -18.3 | 20.8 |
| 70% | 80% | -3.6 | 10.0 | -23.2 | 16.0 |
| 80% | 90% | -16.4 | 10.5 | -36.9 | 4.1 |
| 90% | 100% | -35.0 | 16.4 | -67.1 | -3.0 |
| 100% | 110% | -45.5 | 20.9 | -86.5 | -4.5 |

| Table A5: Ownership of ITNs and/or receipt of IRS by 2010 and total household spending (log) in 2010 | | | | | | | | | | | | | | | | |
| --- | --- | --- | --- | --- | --- | --- | --- | --- | --- | --- | --- | --- | --- | --- | --- | --- |
| Treatment level | Treatment level + 10 percentage points | | | Treatment Effect | | | | Standard Error | | | Lower bound | | | Upper bound | |  |
| 0.1 | 0.2 | | | 0.22 | | | | 0.19 | | | -0.14 | | | 0.59 | |  |
| 0.2 | 0.3 | | | 0.23 | | | | 0.18 | | | -0.13 | | | 0.58 | |  |
| 0.3 | 0.4 | | | 0.20 | | | | 0.20 | | | -0.20 | | | 0.60 | |  |
| 0.4 | 0.5 | | | 0.10 | | | | 0.36 | | | -0.60 | | | 0.80 | |  |
| 0.5 | 0.6 | | | 0.04 | | | | 0.36 | | | -0.66 | | | 0.75 | |  |
| 0.6 | 0.7 | | | 0.07 | | | | 0.30 | | | -0.51 | | | 0.65 | |  |
| 0.7 | 0.8 | | | 0.03 | | | | 0.33 | | | -0.62 | | | 0.68 | |  |
| 0.8 | 0.9 | | | -0.09 | | | | 0.20 | | | -0.48 | | | 0.30 | |  |
| 0.9 | 1 | | | -0.20 | | | | 0.18 | | | -0.56 | | | 0.16 | |  |
| 1 | 1.1 | | | -0.25 | | | | 0.31 | | | -0.85 | | | 0.35 | |  |
|  |  | | |  | | | |  | | |  | | |  | |  |
| Table A6a: Ownership of ITNs and/or receipt of IRS by 2010 and total food spending (log) in 2010 | | | | | | | | | | | | | |  |  |  |
| Treatment level | Treatment level + 10 percentage points | | Treatment Effect | | Standard Error | | | | | Lower bound | | | Upper bound |  |  |  |
| 0.1 | 0.2 | | 0.19 | | 0.14 | | | | | -0.09 | | | 0.46 |  |  |  |
| 0.2 | 0.3 | | 0.18 | | 0.21 | | | | | -0.23 | | | 0.60 |  |  |  |
| 0.3 | 0.4 | | 0.16 | | 0.15 | | | | | -0.14 | | | 0.46 |  |  |  |
| 0.4 | 0.5 | | 0.08 | | 0.28 | | | | | -0.48 | | | 0.64 |  |  |  |
| 0.5 | 0.6 | | 0.03 | | 0.19 | | | | | -0.34 | | | 0.41 |  |  |  |
| 0.6 | 0.7 | | 0.04 | | 0.13 | | | | | -0.22 | | | 0.30 |  |  |  |
| 0.7 | 0.8 | | 0.01 | | 0.17 | | | | | -0.33 | | | 0.35 |  |  |  |
| 0.8 | 0.9 | | -0.08 | | 0.15 | | | | | -0.38 | | | 0.21 |  |  |  |
| 0.9 | 1 | | -0.17 | | 0.14 | | | | | -0.44 | | | 0.10 |  |  |  |
| 1 | 1.1 | | -0.21 | | 0.14 | | | | | -0.49 | | | 0.08 |  |  |  |
|  |  | |  | |  | | | | |  | | |  |  |  |  |
| Table A6b: Ownership of ITNs and/or receipt of IRS by 2010 and total food spending (log) (removing outliers) in 2010 | | | | | | | | | | | | | |  |  |  |
| Treatment level | Treatment level + 10 percentage points | | Treatment Effect | | | | Standard Error | | Lower bound | | | Upper bound | |  |  |  |
| 0.1 | 0.2 | | 0.15 | | | | 0.13 | | -0.10 | | | 0.41 | |  |  |  |
| 0.2 | 0.3 | | 0.16 | | | | 0.11 | | -0.07 | | | 0.38 | |  |  |  |
| 0.3 | 0.4 | | 0.13 | | | | 0.15 | | -0.16 | | | 0.43 | |  |  |  |
| 0.4 | 0.5 | | 0.06 | | | | 0.25 | | -0.42 | | | 0.55 | |  |  |  |
| 0.5 | 0.6 | | 0.02 | | | | 0.21 | | -0.38 | | | 0.43 | |  |  |  |
| 0.6 | 0.7 | | 0.04 | | | | 0.20 | | -0.35 | | | 0.43 | |  |  |  |
| 0.7 | 0.8 | | 0.02 | | | | 0.22 | | -0.41 | | | 0.46 | |  |  |  |
| 0.8 | 0.9 | | -0.05 | | | | 0.14 | | -0.33 | | | 0.23 | |  |  |  |
| 0.9 | 1 | | -0.12 | | | | 0.16 | | -0.42 | | | 0.19 | |  |  |  |
| 1 | 1.1 | | -0.15 | | | | 0.19 | | -0.51 | | | 0.22 | |  |  |  |
| Table A7a: Ownership of ITNs and/or receipt of IRS by 2010 and probability of being in school (for school-aged children) in 2010 | | | | | | | | | | | | | | |  |  |
| Treatment level | | Treatment level + 10 percentage points | Treatment Effect | | | Standard Error | | | | Lower bound | | | | Upper bound |  |  |
| 0.1 | | 0.2 | -0.01 | | | 0.02 | | | | -0.04 | | | | 0.03 |  |  |
| 0.2 | | 0.3 | 0.00 | | | 0.02 | | | | -0.03 | | | | 0.04 |  |  |
| 0.3 | | 0.4 | 0.01 | | | 0.02 | | | | -0.03 | | | | 0.05 |  |  |
| 0.4 | | 0.5 | 0.01 | | | 0.02 | | | | -0.03 | | | | 0.05 |  |  |
| 0.5 | | 0.6 | 0.01 | | | 0.03 | | | | -0.04 | | | | 0.06 |  |  |
| 0.6 | | 0.7 | 0.01 | | | 0.03 | | | | -0.05 | | | | 0.06 |  |  |
| 0.7 | | 0.8 | 0.00 | | | 0.02 | | | | -0.04 | | | | 0.04 |  |  |
| 0.8 | | 0.9 | -0.01 | | | 0.01 | | | | -0.04 | | | | 0.01 |  |  |
| 0.9 | | 1 | -0.02 | | | 0.02 | | | | -0.06 | | | | 0.03 |  |  |
| 1 | | 1.1 | -0.01 | | | 0.03 | | | | -0.07 | | | | 0.04 |  |  |
|  | |  |  | | |  | | | |  | | | |  |  |  |
| Table A7b: Ownership of ITNs and/or receipt of IRS by 2010 and years of schooling in 2010 (for school aged respondents or older) in 2010 | | | | | | | | | | | | | | |  |  |
| Treatment level | | Treatment level + 10 percentage points | Treatment Effect | | | Standard Error | | | | Lower bound | | | | Upper bound |  |  |
| 0.1 | | 0.2 | 0.49 | | | 0.39 | | | | -0.26 | | | | 1.25 |  |  |
| 0.2 | | 0.3 | 0.58 | | | 0.42 | | | | -0.25 | | | | 1.41 |  |  |
| 0.3 | | 0.4 | 0.54 | | | 0.48 | | | | -0.40 | | | | 1.48 |  |  |
| 0.4 | | 0.5 | 0.27 | | | 0.43 | | | | -0.58 | | | | 1.11 |  |  |
| 0.5 | | 0.6 | 0.07 | | | 0.46 | | | | -0.84 | | | | 0.98 |  |  |
| 0.6 | | 0.7 | 0.13 | | | 0.52 | | | | -0.88 | | | | 1.15 |  |  |
| 0.7 | | 0.8 | 0.15 | | | 0.89 | | | | -1.60 | | | | 1.89 |  |  |
| 0.8 | | 0.9 | 0.00 | | | 0.35 | | | | -0.68 | | | | 0.68 |  |  |
| 0.9 | | 1 | -0.15 | | | 0.47 | | | | -1.07 | | | | 0.78 |  |  |
| 1 | | 1.1 | -0.19 | | | 0.83 | | | | -1.82 | | | | 1.45 |  |  |

| Table A8a: Ownership of ITNs and/or IRS by 2008 and total maize production (kg) in 2009 | | | | | | |  |
| --- | --- | --- | --- | --- | --- | --- | --- |
| Treatment level | Treatment level + 10 percentage points | Treatment Effect | | Standard Error | | Lower bound | Upper bound |
| 0.1 | 0.2 | 107.9 | | 101.5 | | -91.0 | 306.8 |
| 0.2 | 0.3 | 100.3 | | 105.8 | | -107.0 | 307.6 |
| 0.3 | 0.4 | 37.2 | | 126.1 | | -210.0 | 284.4 |
| 0.4 | 0.5 | -22.0 | | 84.2 | | -187.0 | 143.0 |
| 0.5 | 0.6 | -45.4 | | 66.3 | | -175.4 | 84.6 |
| 0.6 | 0.7 | -63.3 | | 80.8 | | -221.6 | 95.1 |
| 0.7 | 0.8 | -41.7 | | 83.8 | | -205.9 | 122.6 |
| 0.8 | 0.9 | 7.4 | | 97.2 | | -183.1 | 197.8 |
| 0.9 | 1 | 33.8 | | 104.5 | | -171.0 | 238.7 |
| 1 | 1.1 | 51.3 | | 122.9 | | -189.5 | 292.1 |
|  |  |  | |  | |  |  |
| Table A8b: Ownership of ITNs and/or IRS by 2008 and total potato production (kg) in 2009 | | | | | | |  |
| Treatment level | Treatment level + 10 percentage points | Treatment Effect | | Standard Error | | Lower bound | Upper bound |
| 0.1 | 0.2 | 0.6 | | 15.8 | | -30.3 | 31.6 |
| 0.2 | 0.3 | -2.4 | | 12.5 | | -26.9 | 22.2 |
| 0.3 | 0.4 | -3.0 | | 9.7 | | -21.9 | 16.0 |
| 0.4 | 0.5 | 1.1 | | 21.2 | | -40.5 | 42.7 |
| 0.5 | 0.6 | -0.5 | | 17.5 | | -34.7 | 33.7 |
| 0.6 | 0.7 | 2.2 | | 8.4 | | -14.2 | 18.6 |
| 0.7 | 0.8 | 2.9 | | 8.7 | | -14.1 | 19.8 |
| 0.8 | 0.9 | -2.8 | | 9.5 | | -21.4 | 15.9 |
| 0.9 | 1 | -6.9 | | 11.7 | | -29.8 | 16.0 |
| 1 | 1.1 | -10.3 | | 11.2 | | -32.1 | 11.6 |
|  |  |  | |  | |  |  |
| Table A8c: Ownership of ITNs and/or IRS by 2008 and total nuts production (kg) in 2009 | | | | | | |  |
| Treatment level | Treatment level + 10 percentage points | | Treatment Effect | | Standard Error | Lower bound | Upper bound |
| 0.1 | 0.2 | | -5.7 | | 10.9 | -27.1 | 15.8 |
| 0.2 | 0.3 | | -6.3 | | 11.4 | -28.6 | 16.1 |
| 0.3 | 0.4 | | -5.1 | | 7.5 | -19.9 | 9.7 |
| 0.4 | 0.5 | | -1.7 | | 10.2 | -21.7 | 18.3 |
| 0.5 | 0.6 | | 0.4 | | 10.0 | -19.2 | 20.0 |
| 0.6 | 0.7 | | 1.6 | | 11.8 | -21.5 | 24.8 |
| 0.7 | 0.8 | | 1.7 | | 10.3 | -18.5 | 21.8 |
| 0.8 | 0.9 | | -2.5 | | 12.3 | -26.6 | 21.5 |
| 0.9 | 1 | | -7.2 | | 11.1 | -28.9 | 14.5 |
| 1 | 1.1 | | -11.2 | | 12.9 | -36.5 | 14.1 |

Table A9: Malaria Control Coverage in 2006 and 2010

| **Province** | **District** | **ITN and/or IRS coverage 2006** | **ITN and/or IRS coverage 2010** | **Percent change** | **IRS coverage 2006** | **IRS coverage 2010** | **Percent change** | **ITN coverage 2006** | **ITN coverage 2010** | **Percent change** |
| --- | --- | --- | --- | --- | --- | --- | --- | --- | --- | --- |
| Central | Chibombo | 0.34 | 0.90 | 164% | 0.04 | 0.04 | -18% | 0.34 | 0.90 | 164% |
|  | Kabwe | 0.84 | 0.94 | 11% | 0.79 | 0.51 | -36% | 0.34 | 0.78 | 134% |
|  | Kapiri-Mposhi | 0.62 | 0.70 | 14% | 0.00 | 0.00 | 0% | 0.62 | 0.70 | 14% |
|  | Mkushi | 0.67 | 0.69 | 3% | 0.00 | 0.04 | . | 0.67 | 0.66 | -1% |
|  | Mumbwa | 0.67 | 0.63 | -6% | 0.00 | 0.22 | . | 0.67 | 0.60 | -10% |
|  | Serenje | 0.48 | 0.76 | 59% | 0.02 | 0.00 | -100% | 0.48 | 0.76 | 59% |
| Copperbelt | Chililabombwe | 0.50 | 0.72 | 45% | . | 0.47 | . | . | 0.48 | . |
|  | Chingola | 0.72 | 0.70 | -3% | 0.63 | 0.35 | -44% | 0.37 | 0.51 | 38% |
|  | Kalulushi | 0.43 | 0.88 | 103% | 0.35 | 0.69 | 98% | 0.24 | 0.72 | 200% |
|  | Kitwe | 0.75 | 0.84 | 12% | 0.66 | 0.62 | -5% | 0.34 | 0.71 | 109% |
|  | Luanshya | 0.34 | 0.63 | 84% | 0.05 | 0.45 | 778% | 0.34 | 0.37 | 7% |
|  | Lufwanyama | 0.64 | 0.90 | 40% | . | 0.31 | . | . | 0.86 | . |
|  | Masaiti | 0.05 | 0.72 | 1475% | 0.00 | 0.44 | . | 0.05 | 0.56 | 1126% |
|  | Mpongwe | 0.10 | 0.95 | 893% | 0.00 | 0.40 | . | 0.10 | 0.80 | 735% |
|  | Mufulira | 0.70 | 0.90 | 29% | 0.72 | 0.65 | -10% | 0.36 | 0.72 | 100% |
|  | Ndola | 0.48 | 0.69 | 42% | 0.34 | 0.43 | 28% | 0.35 | 0.52 | 49% |
| Eastern | Chadiza | 0.42 | 0.76 | 82% | 0.00 | 0.00 | . | 0.42 | 0.76 | 82% |
|  | Chama | 0.38 | 0.84 | 124% | 0.00 | 0.00 | . | 0.38 | 0.84 | 124% |
|  | Chipata | 0.47 | 0.83 | 77% | 0.01 | 0.17 | 1478% | 0.47 | 0.79 | 70% |
|  | Katete | 0.10 | 0.81 | 695% | 0.02 | 0.11 | 520% | 0.10 | 0.80 | 688% |
|  | Lundazi | 0.52 | 0.65 | 25% | 0.08 | 0.01 | -82% | 0.48 | 0.65 | 35% |
|  | Mambwe | 0.80 | 0.85 | 5% | . | . | . | . | . | . |
|  | Nyimba | 0.54 | 0.67 | 24% | . | 0.00 | . | . | 0.67 | . |
|  | Petauke | 0.43 | 0.83 | 94% | . | 0.44 | . | . | 0.81 | . |
| Luapula | Chiengi | 0.26 | 0.85 | 226% | 0.00 | 0.00 | . | 0.26 | 0.85 | 226% |
|  | Kawambwa | 0.44 | 0.64 | 44% | 0.00 | 0.50 | . | 0.44 | 0.26 | -40% |
|  | Mansa | 0.38 | 0.56 | 45% | 0.00 | 0.00 | . | 0.38 | 0.56 | 45% |
|  | Milengi | 0.45 | 0.75 | 69% | . | . | . | . | . | . |
|  | Mwense | 0.67 | 0.58 | -13% | 0.00 | 0.00 | . | 0.67 | 0.58 | -13% |
|  | Nchelenge | 0.16 | 0.67 | 317% | 0.00 | 0.37 | . | 0.16 | 0.49 | 206% |
|  | Samfya | 0.41 | 0.40 | -2% | 0.00 | 0.00 | . | 0.41 | 0.40 | -2% |
| Lusaka | Chongwe | 0.27 | 0.85 | 220% | 0.01 | 0.50 | 5453% | 0.27 | 0.71 | 168% |
|  | Kafue | 0.31 | 0.60 | 94% | 0.16 | 0.20 | 23% | 0.23 | 0.49 | 116% |
|  | Luangwa | 0.44 | 0.96 | 118% | . | . | . | . | . | . |
|  | Lusaka | 0.35 | 0.62 | 76% | 0.13 | 0.39 | 193% | 0.27 | 0.46 | 68% |
| North Western | Chavuma | 0.67 | 0.61 | -9% | 0.00 | 0.02 | . | 0.67 | 0.61 | -9% |
|  | Kabompo | 0.23 | 0.92 | 294% | . | 0.00 | . | . | 0.92 | . |
|  | Kasempa | 0.47 | 0.69 | 45% | 0.00 | . | . | 0.47 | . | . |
|  | Mufumbwe | 0.44 | 0.78 | 77% | 0.00 | 0.00 | . | 0.44 | 0.78 | 77% |
|  | Mwinilunga | 0.30 | 0.92 | 208% | 0.01 | 0.00 | -1% | 0.30 | 0.92 | 208% |
|  | Solwezi | 0.35 | 0.72 | 108% | 0.00 | 0.26 | . | 0.35 | 0.55 | 57% |
|  | Zambezi | 0.76 | 0.68 | -11% | . | 0.00 | . | . | 0.68 | . |
| Northern | Chilubi | 0.33 | 0.50 | 51% | . | 0.12 | . | . | 0.43 | . |
|  | Chinsali | 0.48 | 0.58 | 20% | 0.00 | 0.02 | . | 0.48 | 0.58 | 20% |
|  | Isoka | 0.21 | 0.64 | 201% | 0.00 | 0.00 | . | 0.21 | 0.64 | 201% |
|  | Kaputa | 0.16 | 0.68 | 319% | 0.00 | 0.00 | . | 0.16 | 0.68 | 319% |
|  | Kasama | 0.35 | 0.72 | 108% | 0.22 | 0.08 | -65% | 0.30 | 0.69 | 128% |
|  | Luwingu | 0.12 | 0.25 | 108% | 0.00 | 0.00 | 0% | 0.12 | 0.25 | 108% |
|  | Mbala | 0.08 | 0.71 | 786% | 0.00 | 0.46 | . | 0.08 | 0.52 | 554% |
|  | Mpika | 0.35 | 0.86 | 148% | 0.00 | 0.38 | . | 0.35 | 0.76 | 120% |
|  | Mporokoso | 0.13 | 0.68 | 444% | 0.00 | 0.00 | 0% | 0.13 | 0.68 | 444% |
|  | Mpulungu | 0.14 | 0.79 | 481% | 0.04 | 0.03 | -28% | 0.09 | 0.75 | 725% |
|  | Mungwi | 0.17 | 0.76 | 356% | 0.00 | 0.00 | 0% | 0.17 | 0.76 | 356% |
|  | Nakonde | 0.36 | 0.70 | 93% | 0.00 | 0.00 | 0% | 0.36 | 0.70 | 93% |
| Southern | Choma | 0.35 | 0.76 | 119% | 0.00 | 0.33 | . | 0.35 | 0.60 | 75% |
|  | Gwembe | 0.42 | 0.96 | 129% | . | 0.00 | . | . | 0.96 | . |
|  | Itezhi-tezhi | 0.72 | 0.75 | 4% | 0.00 | . | . | 0.72 | . | . |
|  | Kalomo | 0.89 | 0.50 | -44% | 0.00 | 0.01 | . | 0.89 | 0.48 | -46% |
|  | Kazungula | 0.76 | 0.79 | 4% | . | 0.51 | . | . | 0.54 | . |
|  | Livingstone | 0.96 | 0.89 | -7% | 0.76 | 0.70 | -7% | 0.67 | 0.64 | -4% |
|  | Mazabuka | 0.36 | 0.87 | 143% | 0.02 | 0.19 | 650% | 0.36 | 0.85 | 137% |
|  | Monze | 0.23 | 0.78 | 244% | 0.00 | 0.25 | . | 0.23 | 0.70 | 210% |
|  | Namwala | 0.25 | 0.96 | 284% | . | 0.00 | . | . | 0.96 | . |
|  | Siavonga | 0.63 | 0.20 | -68% | . | 0.00 | . | . | 0.20 | . |
|  | Sinazongwe | 0.46 | 0.80 | 75% | 0.00 | . | . | 0.46 | . | . |
| Western | Kalabo | 0.96 | 0.50 | -48% | 0.00 | 0.00 | 0% | 0.96 | 0.50 | -48% |
|  | Kaoma | 0.16 | 0.74 | 360% | 0.00 | 0.30 | . | 0.16 | 0.68 | 322% |
|  | Lukulu | 0.65 | 0.75 | 15% | 0.00 | 0.00 | 0% | 0.65 | 0.75 | 15% |
|  | Mongu | 0.86 | 0.89 | 3% | 0.11 | 0.26 | 138% | 0.86 | 0.86 | 0% |
|  | Senanga | 0.89 | 0.96 | 7% | 0.00 | 0.77 | . | 0.89 | 0.83 | -7% |
|  | Sesheke | 0.60 | 0.61 | 1% | 0.00 | 0.00 | 0% | 0.60 | 0.61 | 1% |
|  | Shang’ombo | 0.60 | 1.00 | 67% | 0.00 | 0.00 | 0% | 0.60 | 1.00 | 67% |

Source is 2006 and 2010 MIS. IRS coverage is percentage of households in the district sprayed over the last 12 months, not the percentage of targeted households. ITN coverage is defined as the proportion of households with at least one ITN.
