# Supplementary material for: Exploring the use of routinely-available, retrospective data to study the association between malaria control scale-up and micro-economic outcomes in Zambia
Source: Malar J. 2017 Jan 4;16:15. doi: 10.1186/s12936-016-1665-z (PMC5209918; doi:10.1186/s12936-016-1665-z)
Supplement: Supplementary file 2 — Additional file 2. Additional figures presenting results with ownership treatment variable. [file 12936_2016_1665_MOESM2_ESM.docx]

**Additional Figures Presenting Results with Ownership Treatment Variable**

Graph A1: Ownership of ITNs and/or IRS by 2010and ln(HH food spending) in 2010 – main result

Graph A2: Ownership of ITNs and/or IRS by 2010and ln(HH food spending) in 2010 – Using Box-Cox transformation of treatment variable

Graph A3: Ownership of ITNs and/or IRS by 2010and ln(HH food spending) in 2010 – Testing GPS score within 3 categories rather than 2 within each bucket

Graph A4: Ownership of ITNs and/or IRS by 2010and ln(HH food spending) in 2010 – Testing different cut points for Treatment interval

Graph A5: Use of ITNs and/or IRS by 2010and ln(HH food spending) in 2010 – main results

Graph A6: Use of ITNs and/or IRS by 2010and ln(HH food spending) in 2010 – allowing use variable to exceed 100%

Graph A7: Use of ITNs and/or IRS by 2010and ln(total HH expenditures) in 2010

Graph A8: Use of ITNs and/or IRS by 2010and ln(HH food spending) in 2010 removing outliers for food spending in 2010

Graph A9a: Use of ITNs and/or IRS by 2010 and probability of being in school (for school-aged children) in 2010

Graph A9b: Ownership of ITNs and/or IRS by 2010 and years of schooling in 2010 (for school aged respondents or older) in 2010

Graph A10a: Use of ITNs and/or IRS by 2008 and total maize production (kg) in 2009

Graph A10b: Use of ITNs and/or IRS by 2008 and total potato production (kg) in 2009

Graph A10c: Use of ITNs and/or IRS by 2008 and total nuts production (kg) in 2009
