# Supplementary material for: Exploring the use of routinely-available, retrospective data to study the association between malaria control scale-up and micro-economic outcomes in Zambia
Source: Malar J. 2017 Jan 4;16:15. doi: 10.1186/s12936-016-1665-z (PMC5209918; doi:10.1186/s12936-016-1665-z)

**Additional Figures Presenting Results for Use Treatment Measure and Secondary Microeconomic Outcomes: Wage Labour, Medical Spending, Education Spending, and Borrowing**

Graph A1: Use of ITNs and/or IRS by 2010 and Wage Labour in 2010


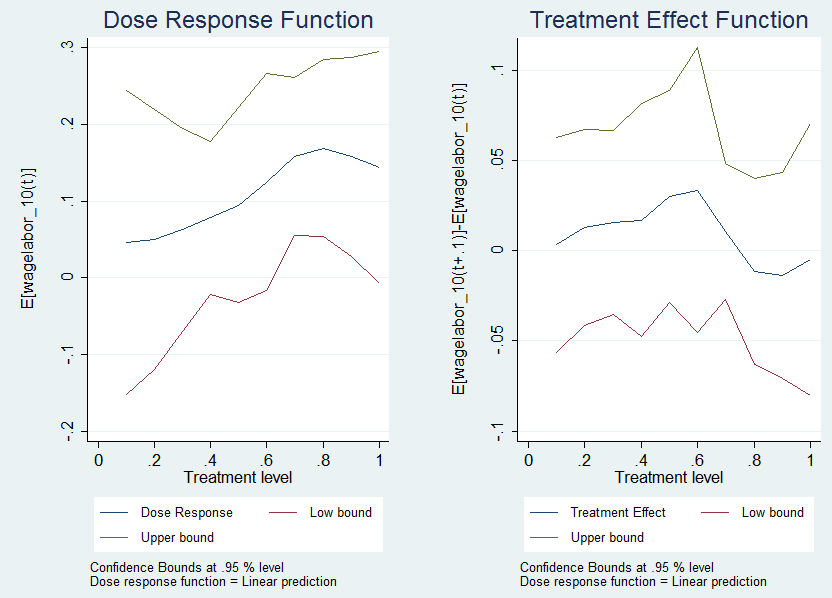


Graph A2: Use of ITNs and/or IRS by 2010 and Medical Spending in 2010


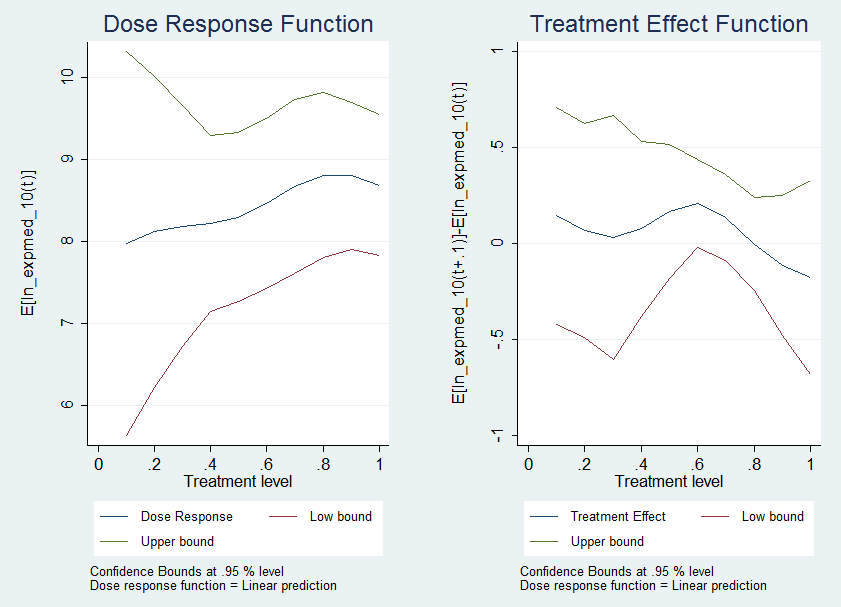


Graph A3: Use of ITNs and/or IRS by 2010 and Education Spending in 2010


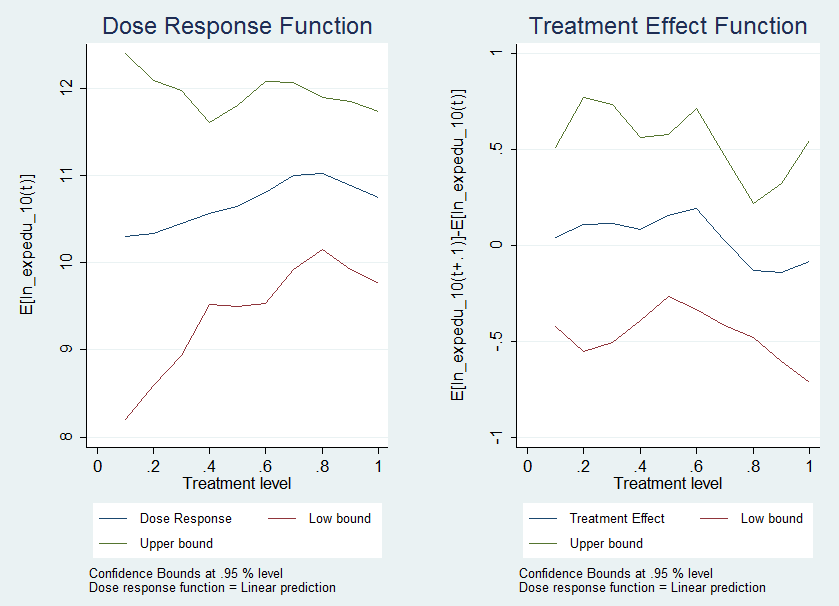


Graph A4: Use of ITNs and/or IRS by 2010 and Household Borrowing in 2010


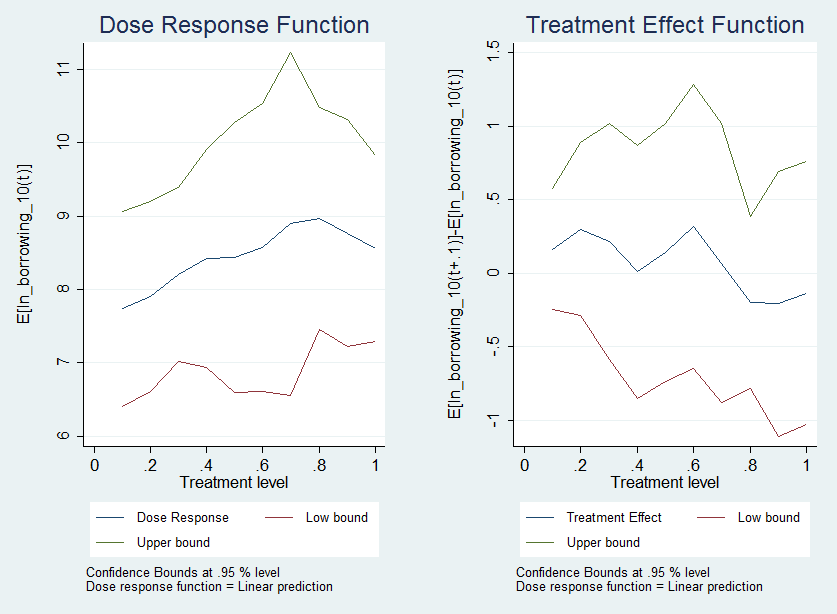

Supplement: Supplementary file 3 — Additional file 3. Additional figures presenting results for use treatment measure and secondary microeconomic outcomes: wage labour, medical spending, education spending, and borrowing. [file 12936_2016_1665_MOESM3_ESM.docx]
